# Simulation study of cone-in-shell target for indirect-drive ion fast ignition concept under the theory of an effective interaction potential

**DOI:** 10.1038/s41598-023-36597-0

**Published:** 2023-06-10

**Authors:** Mahsa Mehrangiz, Soheil Khoshbinfar

**Affiliations:** Department of Physics, Faculty of Sciences, University of Guilan, P.O. Box: 41335-1914, Rasht, Iran

**Keywords:** Nuclear energy, Nuclear fusion and fission

## Abstract

The stopping power of charged particles released by the deuterium–tritium nuclear reactions has been extensively studied in the weakly to moderately coupled plasma regimes. We have modified the conventional effective potential theory (EPT) stopping framework to have a practical connection to investigate the ions energy loss characteristics in fusion plasma. Our modified EPT model differs from the original EPT framework by a coefficient of order $1 + {2 \mathord{\left/ {\vphantom {2 {(5}}} \right. \kern-0pt} {(5}}\ln \overline{\Xi }),$($\ln \overline{\Xi }$ is a velocity-dependent generalization of the Coulomb logarithm). Molecular dynamics simulations agree well with our modified stopping framework. To study the role of related stopping formalisms in ion fast ignition, we simulate the cone-in-shell configuration under laser-accelerated aluminum beam incidence. In ignition/burn phase, the performance of our modified model is in agreement with its original form and the conventional Li-Petrasso (LP) and Brown-Preston-Singleton (BPS) theories. The LP theory indicates the fastest rate in providing ignition/burn condition. Our modified EPT model with a discrepancy of $\sim$ 9%, has the most agreement with LP theory, while that of the original EPT (with a discrepancy of $\sim$ 47% to LP) and BPS (with a discrepancy of $\sim$ 48% to LP) methods maintain the third and fourth contributions in accelerating the ignition time, respectively.

## Introduction

In a strongly coupled plasma, such as we have in the inertial confinement fusion (ICF), there have been some processes, including diffusion or temperature relaxation occur that require a deep understanding of the complex plasma system^1^. Moreover, the screening or correlation effects of the plasma components are present^2,3^. In this case, a classical one-component plasma (OCP) is considered, where a specified projectile moves in the presence of an inert neutralized background. Its energy is studied using molecular-dynamics (MD) simulations. Despite the fact that the effects of strong Coulomb coupling are included in an OCP, electron physics and multiple species in the dense plasma have not been considered^4–7^.

Recent experimental studies indicate that the stopping power of ions propagating in hot dense plasma regimes, support the predictions of Li-Petrasso (LP) and Brown-Preston-Singleton (BPS) analytical stopping power formalisms^8–10^. However, these two commonly accepted models are not appropriate in dielectric response. They operate within the weakly to moderately coupled plasma regimes. While in strongly coupled plasmas, the quantum mechanical based-methods, such as ab initio time-dependent orbital-free density functional theory (TD-of-DFT) provides a more precise charged particle stopping model^11–13^. Ding et al. showed that the use of ab initio TD-of-DFT theory with the assumption of deuterium–tritium (DT) produced alpha particles may have resulted in lowering the stopping power up to 25% when compared with the conventional stopping frameworks used in the high-energy–density plasmas (HEDP)^14^. Furthermore, considering the extensive path-integral Monte Carlo data, Groth, Dornheim, and colleagues confirmed the results for the dynamic density response of the electron gas in a warm-dense-matter (WDM) regime^15,16^. More recently, by developing the ab initio quantum Monte Carlo (QMC) machine-learning representation, Moldabekov et al. focused on the polarization-induced stopping power due to the struggling rate, free electrons and friction functions to investigate the charged particles stopping power in non-ideal dense plasmas^17^.

In 2014, Baalrud and Daligault proposed a new theory known as an effective-potential-theory (EPT) to extend the plasma transport theory from weakly to strongly coupled plasma regimes^7,18^. They have derived an expression for the transport coefficient by Taylor’s expansion of the Fokker–Planck (FP)-based collision operator. Their model may be applied to calculate the stopping power of the incident charged particles in target plasma. In this theory, the particle interactions occur via the potential of mean force^18,19^. In addition, the excluded volume in repulsive interactions is considered to implement a modified version of Enskog’s kinetic equation for hard spheres^18^. Validation from experiments and MD simulations has shown EPT model is reasonably accurate with the possible exception to the liquid-like correlation parameters, with the coupling strength, Γ (i.e. the ratio of the Coulomb energy to the thermal one), of approximately 10–50, for OCP^18^. They have also concluded that the EPT-based predictions versus FP forms of the kinetic equation are likely to lead to similar predictions for the transport coefficient.

The basis of the plasma transport physical consideration is the Coulomb collision calculations. The cumulative effects of these collisions i.e. the known Coulomb logarithm, lnΛ, is the key factor. This quantity, which is the measure of the small-angle collisions to large-angle scattering becomes more important at the intermediate to strongly coupled plasma regimes. In LP stopping formalism, it is emphasized the importance of large-angle scattering as well as the small-angle collisions in moderately coupled plasmas (2 ≤ lnΛ ≤ 10) suitable for the igniting of DT plasma in ICF^8,9^. It is equivalent to an explicit contribution of the Coulomb logarithm in the collision operator. Therefore, in contrast with the Rosenbluth’s original treatment of the FP equation^20^, they generalized the FP equation retaining the third and parts of the second-order-terms in the Taylor expansion of the collision operator to have an adequate justification in lnΛ ≥ 2 plasmas. The effects of large-angle scattering have also great importance in a strongly coupled plasmas (i.e. lnΛ ≤ 1). Here, owing to the lower values of the Coulomb logarithm, it is expected that the contribution of the 1/lnΛ terms in the collision operator has an unequivocal significance. Thus, considering only the first two terms in EPT stopping formalism, may not bring a realistic picture of the charged particles energy loss in a strongly coupled plasma. The latter has a precise significance in the ICF plasma regime during the compression and subsequently, the ignition and burn phases, which are currently pursued at the national ignition facility (NIF)^21,22^.

Albeit the national ignition facility, is designed to analyze the ICF in the indirect-drive configuration, no indirect-drive integrated fast ignition (FI) or ion fast ignition (IFI) experimental research has been carried out yet. While, the FI and specifically IFI schemes have been of great interest to reach a high gain in ICF recent research. The lack of an appropriate intensive laser pulse, mixing between the ablated gold and the compressed fuel core, and the generation and acceleration of collimated quasi-monoenergetic ion beams and their efficient propagation within the guide cone and hohlraum regard as the main obstacles^23,24^. Taking into account these complexities, one can obtain computer simulations as good references to understand the dynamic of acceleration mechanisms, implosion, ignition and burn phases of fuel plasma, experimental targets, and setup configuration. Heretofore, one-dimensional and two-dimensional numerical simulations have been extensively used due to their limited requirement to compute initiatives.

For the scheme of IFI, the ion beam intensity will be extremely high. It was shown that the propagation of fast ions through matter, such as plasma medium, shows a medium “density effect”, where collective polarization of the medium produces a partial cancellation of the fields of the fast ions, reducing the energy loss rate by the ion^25,26^. Therefore, it would remarkably affect the ion stopping process because when the beam densities are sufficiently high, an enhancement of stopping power occurs^26^.

New experimental analyses are being reported in recent years, including the stopping of fast particles in ICF-relevant plasmas generated by implosions^27^, warm-dense matter^28,29^, and laser-produced plasmas followed by accelerator beams^30,31^. These experimental measurements span a wide range of different plasma regimes; however, the probing test charges lose a comparatively small fraction of their initial energy. Therefore, a standard and suitable treatment of the stopping formalism is indispensable for analogy to the obtained data and other acceptable theories.

In ICF research, the LP theory is broadly considered due to its perceptual simplicity in computation and analysis. Although from kinetical point of view, BPS regards as another combined slowing-down technique, there is a lack of studies in EPT formalism to prob its applicability to ICF modeling.

The focus of this research is on analyzing the role of three slowing-down models, including LP, BPS, and EPT in fast ignition (FI) of ICF relevant plasma targets. To this aim, we simulate a cone-in-shell target in an indirect-drive IFI scheme. With this simulation, we aim to simultaneously study the acceleration of quasi-monoenergetic ion beam, its interaction with the imploded DT core, and its effect on the ignition and burn phases of core. As another attempt, we also prob the modifications of collision operator to the original EPT model, and compare to other three related stopping theories using in evaluating FI plasma.

## Results

### Theory of a modified link to the original EPT framework

A fast charge slowing-down in a weakly coupled plasma regime is well characterized. However, because ICF relevant experiments are almost moderately coupled and many rely on fuel plasma heating by fast fusion product ions^9,32,33^, there has been an effort to document the stopping framework in dense plasmas with theory and MD simulations^9,32–36^.

The broad use of LP theory in ICF research, and the attractiveness of their technique prompted us to re-analyze their original theory by calculating the fourth term in Taylor expansion (the calculated structural parameters are analyzed in detailed in Supporting Information, Fig. S3). However, the proper reported treatment of EPT framework in weakly to strongly coupled regimes was an incentive for us to study the modifications efforts followed in LP theory to the original EPT in order to evaluate the results.

The FP approach can be obtained from a Taylor series expansion of the Boltzmann equation in terms of the momentum transfer in a collision. The connection between these two approaches in the original EPT model is interpreted in reference 18. The Boltzmann form does not expand in terms of scattering angle like the FP approach does. Therefore, one can get a more accurate answer by computing the stopping power directly from the Boltzmann equation instead of having higher-order-terms of FP results previously probed in LP theory. We have carefully evaluated the latter issue. We found that in a weakly coupled regime, no matter how high of order a FP equation is carried out, it would ultimately converge to the Boltzmann equation (or Boltzmann-like in the case of the mean force kinetic equation). However, spanning from weak to strong coupling, the corrections we see from a higher order FP method would not be very approach to the Boltzmann solution. So that, based on our theoretical calculations, by keeping the fifth number of terms, an improved coefficient,$1 + {2 \mathord{\left/ {\vphantom {2 {(5}}} \right. \kern-0pt} {(5}}\ln \overline{\Xi }),$ in the second-term of expansion will be obtained, where $\ln \overline{\Xi }$ regards as a velocity-dependent generalization of the Coulomb logarithm. The latter can clarify that a high-order FP method would be more accurate than keeping the full Boltzmann that the original EPT model does. The calculation method and obtained parameters are available in the Supporting Information. Note that in the Supporting Information, the parameter $\ln \overline{\Xi }$, is shown by $\ln \overline{\Xi }_{{ss^{\prime}}}$ during the calculations to emphasize that the result obtained by assuming the test particle, *s*, collides with the field particle,$s^{\prime},$ with the velocities ${\mathbf{v}}_{s}$ and ${\mathbf{v}}_{{s^{\prime}}} ,$ respectively.

**Figure 1:**
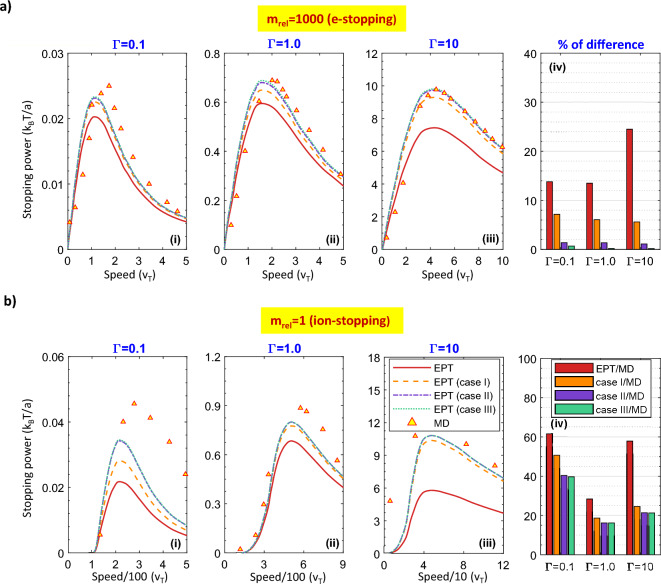
Comparisons of stopping power for m_rel_ = 1000, and m_rel_ = 1 at the coupling strength values, Γ, of 0.1, 1, and 10. Red-solid curve refers to original EPT model; orange-dashed, purple-dash-dotted, and green-dotted curves, respectively introduce the effects of third to fifth orders of FP method to the original EPT framework. MD results are specified with yellow triangles. To further comparison, the percentage difference of each stopping curves to MD results is expressed in the bar graphs for the two relative masses.

As a first comparison, Fig. 1 compares the modified EPT stopping (cases (I–III)) results to the original EPT formalism for two different relative masses. Here, cases (I–III), respectively introduces the addition of third to fifth orders to the original EPT framework predicted in reference 18. To further comparison, we include MD simulation results using the Large-scale Atomic/Molecular Massively Parallel Simulator (LAMMPS) developed by Sandia National Laboratory^37^ as is explained in the Methods. One of the aims of this research is to explore how different coupling strengths can affect stopping power. As Γ increases, one can expect three flagrant changes which can be clarified by examining the friction term of collisional models and their dependance on the generalized Coulomb logarithm,$\ln \overline{\Xi }$. In other words, the $\ln \overline{\Xi }$ parameter includes a term that expresses the physics of collisions for a given relative speed between each two particles, and another that characterizes the probability of those collisions occurring.

The related apparent changes include the speed at which the Bragg peak rises. The slowing-down curve broadens over speed, and as previously declared, the slowing-down curve increases in magnitude (in units of $k_{B} T/a$).

For m_rel_ = 1000, one can see that for the coupling strength value of Γ = 0.1, the formalism based on EPT, cases (I–III), and MD simulation agree well at low speeds. At the Bragg peak, the original EPT model under predicts the stopping curves. At high speeds, the conventional EPT formalism still under predicts the MD results. However, as one can see, the MD results agree well with cases II, and III.

For Γ ≥ 1, both EPT and its modified frameworks qualitatively predict the MD data. However, compared to cases (I–III), the conventional EPT quantitatively under predicts the MD results to a greater degree while cases II and III, have almost the same behavior as the MD data.

Another apparent issue is the Bragg peak broadening and shifting to higher test particle speeds, at strong coupling regimes, which can be caused by the insensitivity of the cross-section factor to the relative velocity of the under-collision particles. On the other hands, at strong coupling, the relative speed of under-collision particles must be fast enough to have scattering angles of almost less than 90°, since the screening effect, limits the range of inter-particle collisions among next neighbors. In that case, the cross section becomes larger in value which can lead to an increase in the normalized slowing-down curves.

The similar conclusions can be made for m_rel_ = 1. Here, at very low speeds, the stopping curve is negative, mentioning the total stopping is dominated by the thermalization term. To better comparison, we also analyzed the percentage difference of the under considered slowing down curves to MD data for different relative masses (Fig. 1aiv and biv). Comparing bars for both relative masses, it reveals that m_rel_ = 1000 can essentially suppress the percentage difference. As a result, if we base our comparison on MD simulation results, we can conclude that increasing the FP orders to the original EPT model shows more agreement in ions-electrons collisions (m_rel_ = 1000). Furthermore, case II has almost the same effect on both relative masses compared with case III which indicates the insignificant effect of FP orders of higher than 5 on the stopping power final results.

As a second comparison, we also compared several slowing-down theories, including LP, BPS, EPT, and its modified framework in an unmagnetized plasma. For simplicity we only considered case II as the modified form of EPT model. The comparison is available in the Supporting Information, Fig. S1 as a function of three different coupling regimes. We illustrated that theories of LP and BPS compare similarly with EPT and case II. However, the results clearly show that LP and BPS stopping curves show poor performance in strongly coupled regimes (Γ = 10).

Our strategy to modify EPT model was to analyze the effect of our efforts on expressing ignition/burn of fusion plasma in cone-guiding FI scenario. In non-ideal conditions, the density and temperature of plasma are not uniform, and this non-uniformity is naturally affected by the inter-particle collisions. Therefore, the rationale for this behavior is to benefit a proper slowing-down model to make well-documented predictions about the ignition/burn condition, especially in hot-spot regime. This hypothesis must be tested by running simulations in which the role of conventional stopping formulations is shown in hydrodynamic evolutions of FI-relevant plasmas. However, the physical processes in ion fast ignition (IFI)- the under-discussion scenario in this paper- are complex. Since they have large spatial ranges, different time scales, propagation of accelerated ions, and multi-physics, it is almost impossible to simulate all the processes within one code.

A typical target for IFI is a shell target fitted with a guide cone to make a pass for the laser-accelerated ion beams. Here, we will simulate and study our hypothesis for indirect-drive IFI technique, using a cone-in-shell target structure inside the hohlraum. We consider the ns laser beams are injected into the hohlraum at an angle of 50^˚^ relative to the hohlraum axis to generate X-ray radiations, which can lead to the CH-DT capsule implosion. The structure of capsule was chosen based on the design followed at the first stage of FIREX-I project^38^. The simulation setup and the structural parameters are available in the Methods.

### Investigation of cone-guided cryogenic target implosion inside the hohlraum

**Figure 2:**
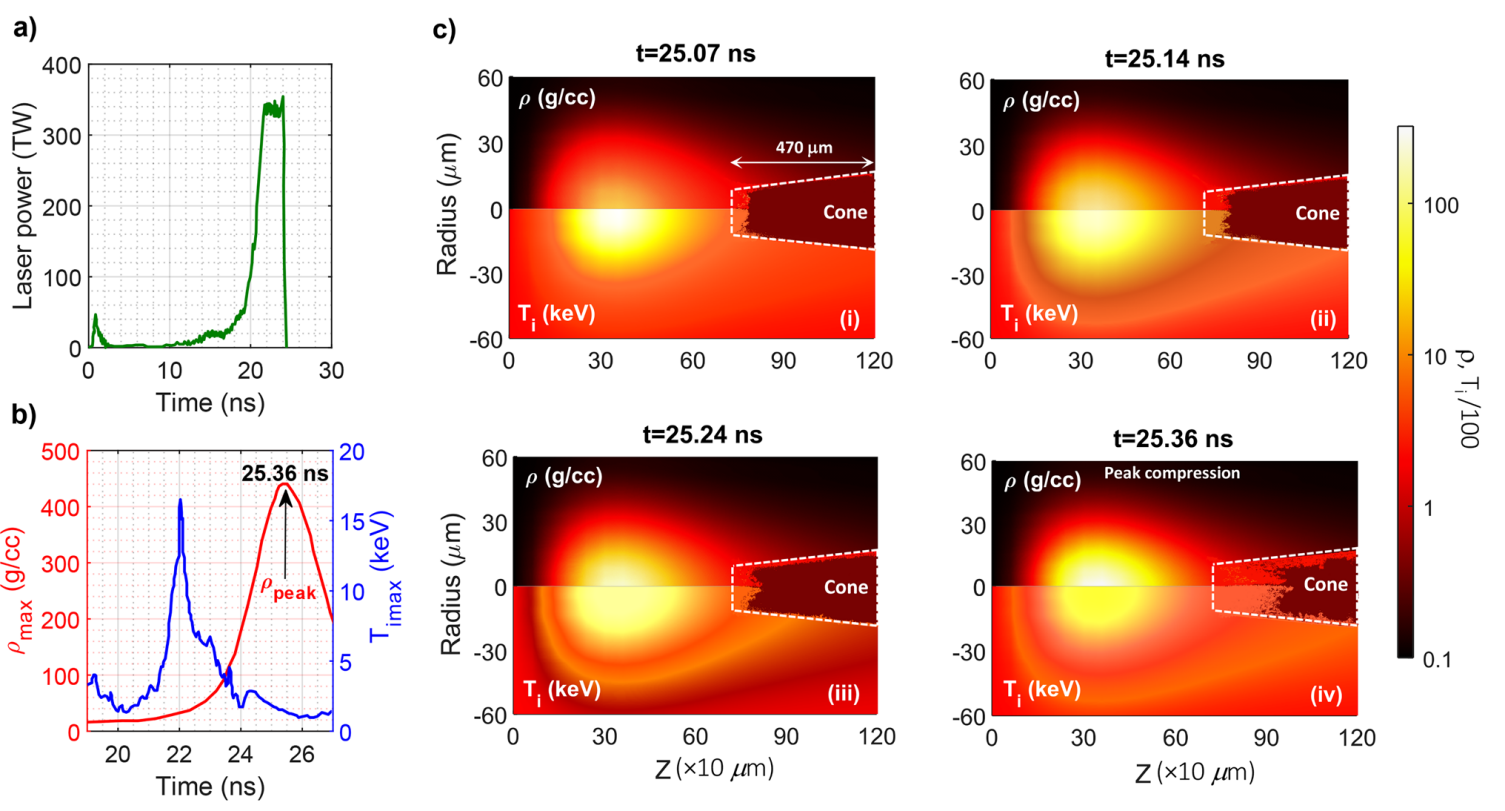
Imploded profiles obtained from MULTI2D simulations for the core-in-shell structure considered in Fig. 5. (**a**) the laser pulse shape considered in simulation for hohlraum drive with a peak power of 354 TW and total energy of 1.314 MJ, (**b**) temporal illustrations of ion maximum temperature (T_imax_) and maximum density (ρ_max_), (**c**) spatial distributions of density and ion temperature at four different capsule-X-ray interaction times. The initial position of the attached cone is shown with white-dashed lines.

Using the schematic illustration introduced in Fig. 5 (see “Methods”), two-dimensional (2D) dynamics of the target implosion is simulated using the radiation hydrodynamic code MULTI2D^39,40^ in Fig. 2 at four different capsule-X-ray interaction times obtained in the simulations for hohlraum drive (see “Modelling”). Note that in order to observe the effect of implosion on guide cone more precisely, we discretize the numerical data and coarsen spatial resolution in *r-z* limits related to the initial position of the cone.

As shown in Fig. 2, in the implosion of cone-in-shell targets, the base capsule shape is far from the spherical one considered just before the interaction. Under these conditions, the cone tip is misshaped by the plasma flow from the imploded core, which gradually increments by increasing the interaction time up to the peak compression (25.36 ns).

From figure, the peak compression is obtained at 25.36 ns. At this time, the most part of the outer shells and the inner parts of capsule are highly compressed. However, the velocity across the interface between the core plasma and deformed cone wall is different, which may lead to the Kelvin–Helmholtz instability occurrence. Therefore, as one can see, the imploded plasma core is rolled up and dragged towards the cone wall. We found that the latter has not much effect on the ionization of the outer walls of the cone, so that the maximum ionization is about the state charges of + 4.

The core density reaches approximately 440.447 g/cc at the maximum compression. Moreover, the pressure imbalance occurred through the existence of the guide cone, leads the hot-spot to move towards the cone tip. At the peak compression, the cone tip notably collapsed and subsequently, the low-density plasma succeeds in forcing a way through the vacuum region inside the cone, which can affect the inner cone walls ionization. According to our obtained results, although the effect of ionization on the inner cone walls is not equal in the four considered implosion times, however, the maximum ionization is approximately + 10 occurred away from the cone tip. Unfortunately, this ionization and the subsequent deformation of cone tip is not preferred for the plasma core heating procedure. Therefore, we aim to follow our further analyses by considering the imploded core structure at 25.24 ns. Note that although the cone tip is already misshaped at this time value, however, the vacuum region inside the guide cone is almost clean (see Fig. 2ciii).

## Core heating by the laser-accelerated aluminum beam

### The aluminum focusing and acceleration simulation

Novel acceleration mechanisms of heavier ion species (Z >  > 1), opened a new set of practical and efficient possibilities in IFI concept^41–44^. However, consideration of difficulties in growing of micro-instabilities occurred through the propagation of heavy ions in core plasma, regards as an indisputable challenge since it can affect the fuel ignition/burn phase. The latter envisioned that the growth rate must be controlled or even damped to acquire enhanced stability. This challenge tested analytically by Khoshbinfar for two different heavy ions of C^6+^ (energy spread $\sim$ 10%) and Al^11+^ (energy spread $\sim$ 20%) at the pre-compressed DT plasma regime^45^. These results declared that in FI by laser-accelerated C/Al ions, benefitting from higher energies (such as those mentioned in reference 45), and subsequently a greater degree of ionization may play a more prominent role in reducing the micro-instabilities arise at the ignition/burn phase of fuel plasma. Therefore, they can be proposed as a suitable alternative for IFI with light ions, such as protons^46–58^.

In order to investigate the effect of laser-accelerated aluminum ions on core heating process, we need to obtain the main characteristics of accelerated ions, such as energy spectra, spatial distribution, and emittance. Aiming at production of high-quality multi-MeV Al ions located at the tip of the conical target illustrated in Fig. 5, We performed the simulation of aluminum ions production using the 2D3V PIC code EPOCH^59^ to find out the beam main parameters. The parameters were then used as the initial values in MULTI code to analyze the ignition/burn phase in cone-in-shell target (see “Methods”).

**Figure 3:**
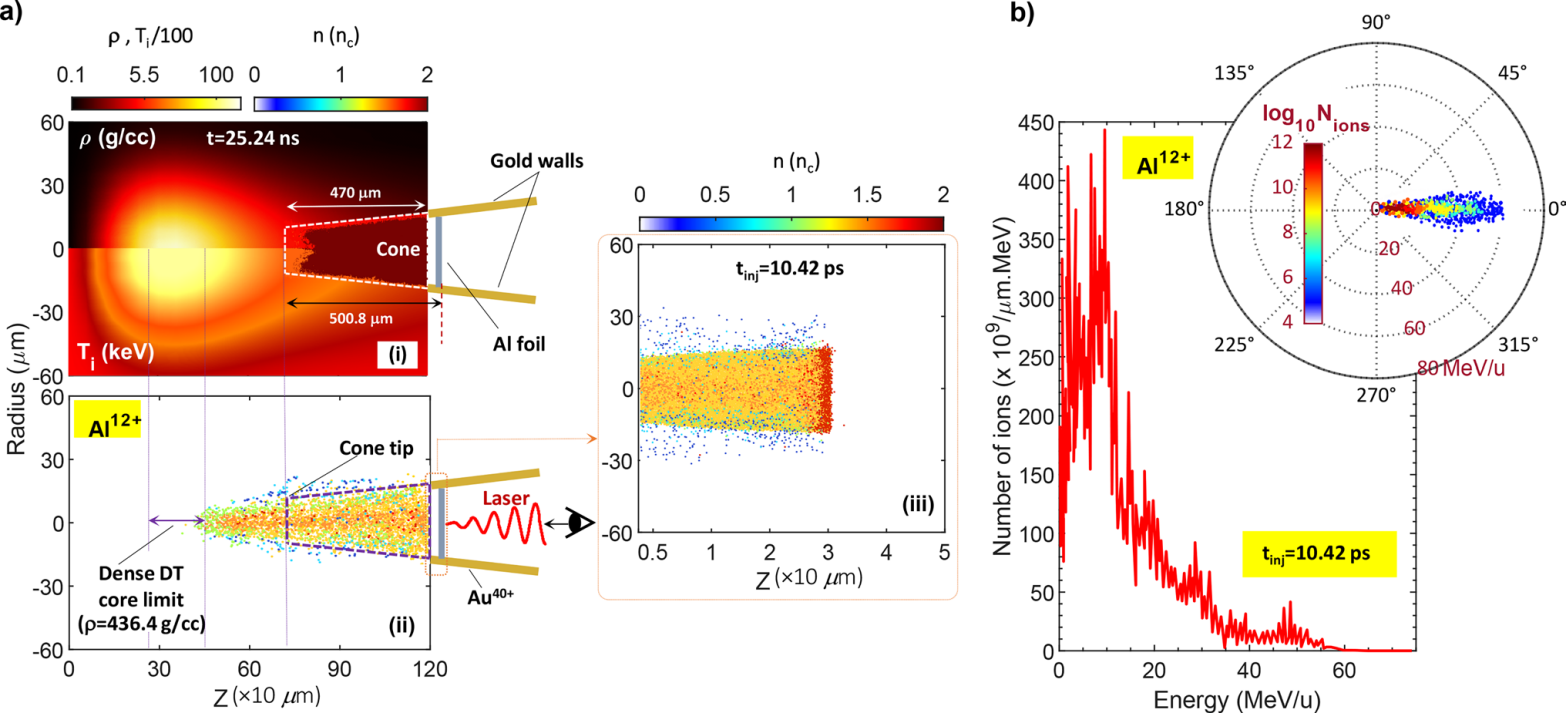
(**a**_**i**_) Spatial distribution of density and ion temperature at 25.24 ns, (**a**_**ii**_) density distribution of Al^12+^ ions from the cone walls and ionized aluminum foil after 10.42 ps of acceleration. To better comparison, the density distribution of the accelerated Al^12+^ ions from the ionized foil is illustrated at the areas outside the imploded core structure simulation box region (see (**a**_**iii**_), (**b**) Al^12+^ ions energy spectra after 10.42 ps of acceleration. The inset shows the ions energy-angle distribution after 10.42 ps of acceleration.

Using the imploded DT core profile at t = 25.24 ns (Fig. 2ciii) as the initial plasma profile (Fig. 3ai), in the rest of this section, we will carry out the core heating simulations after the injection of accelerated aluminum beam. The laser pulse potentially ionizes the cone gold walls and Al foil to state charges of Au^40+^, and Al^12+^, respectively (the details are available in Methods). The Al^12+^ ions density distribution is plotted in Fig. 3aii. Since we are looking to investigate imploded core heating (ignition/burn) after the beam injection, we continued the PIC simulation till the time in which the accelerated Al^12+^ ions have reached the peak compression region of imploded core (t_inj_ = 10.42 ps). As schematically plotted in Fig. 3ai and aii, the Al-foil is located outside of the initial implosion simulation box. Therefore, we also illustrated the missed spatial distribution of Al-beam from the ionized foil in Fig. 3aiii. From Fig. 3a, one can see that the Al^12+^ ions density distribution includes a more compact concentration around the central region. However, accelerated ions become very compact at *z* $\sim$ 470 μm, indicating that Al^12+^ ions have possessed a high-density distribution for distances far from the cone tip. In fact, the transverse electric field produced by the fast electrons induced by the charge separation in acceleration regime, trying to propagate through the cone walls interface. However, as Au^40+^ ions are much heavier than Al^12+^ ions, their acceleration towards the cone axis is not as serious. In this case, the hot electrons are trapped in the cone walls, generating a surface current towards the cone tip, which can push the accelerated Al^12+^ ions towards the cone axis and compacting them in a diameter smaller than the cone tip (see Fig. 3aii). Similar to this case has been discussed earlier in reference 58.

To better analyzation, we have depicted the Al^12+^ ions energy spectra at the injection time (10.42 ps). From figure, the peak is well expressed and dispersion is decreased. As shown in the inset, the acceleration scheme presents a high-quality aluminum bunch with the opening angle of *θ* $\sim$ 5.6° just at the beam-core injection time. At this time, the accelerated ions maintain the cut-off energy of approximately 60 MeV/u. Moreover, the maximum number of accelerated Al^12+^ ions is about 4.48 × 10^11^/μm.MeV corresponds to the energy of around 10 MeV/u. As a precise determination of the beam characteristics required for DT core heating, we computed the average energy, E_ave,Al_, and energy spread, ΔE_Al_/E_Al_, of Al^12+^ ions, which were calculated about 17 MeV/u and 0.24, respectively.

### Ignition condition for the imploded DT plasma configuration

In order to ignite an imploded fuel, the incident beam must be irradiated the target at the times of less than the hydrodynamic equilibrium time (τ_eq_)^60^. Otherwise, as the plasma target expands, it will not have the density required to initiate ignition procedure. In our previous research followed in reference 60, we showed that for a spherical DT hot-spot with an initial radius and temperature of 20 μm and 1 keV, respectively, the estimated hydrodynamic equilibrium time will be at least 26 ps.

In the current research, our simulation results in Fig. 2 illustrated an almost elliptical profile for DT core with the semi-minor axis and semi-major axis of about 18.9 μm and 27.2 μm, respectively. We also investigated the ignition energy threshold, E_ig_, for this DT core profile driven by an aluminum beam. Dependently of the four considered stopping formalisms, we found the average value of about E_ig,ave_ = 8.29 kJ for imploded DT plasma at injection time. The calculated structural parameters are available in Methods Fig. 6.

To more accuracy, we calculated the deposition radius for the under-consideration stopping frameworks. The calculated radiuses are 4.86 μm (for LP), 5.48 μm (for EPT (case II)), 6.08 μm (for conventional EPT), and 7 μm (for BPS). In an effort done by Roth et al. he suggested that the required optimal focal spot radius must be approximately $\le 60/\left[ {\rho /(100g/cc)} \right]^{0.97} \mu m$ for the collimated proton ignitor beam^48^. In accordance with this result, if our current dense DT core (436.4 g/cc) ignited by fast protons, we expected the required beam radius of at least 14.4 μm. Nonetheless, the related value expects to be suppressed for quasi-monoenergetic aluminum beam due to its lower charge to mass ratio. As discussed, this claim is in agreement with the calculated values of beam radius for considered stopping formalisms.

Under these assumptions, the hydrodynamic equilibrium time can be considered as at least 30 ps. In this case, having the ignition energies introduced in Methods (Fig. 6), we assume that the injected aluminum beam irradiates the imploded core for 23 ps. In addition, as an initial condition, we consider that the pre-compressed equimolar DT hot-spot maintains the initial temperature and density of approximately 1.52 keV and 436.4 g/cc, respectively (see Fig. 2).

**Figure 4:**
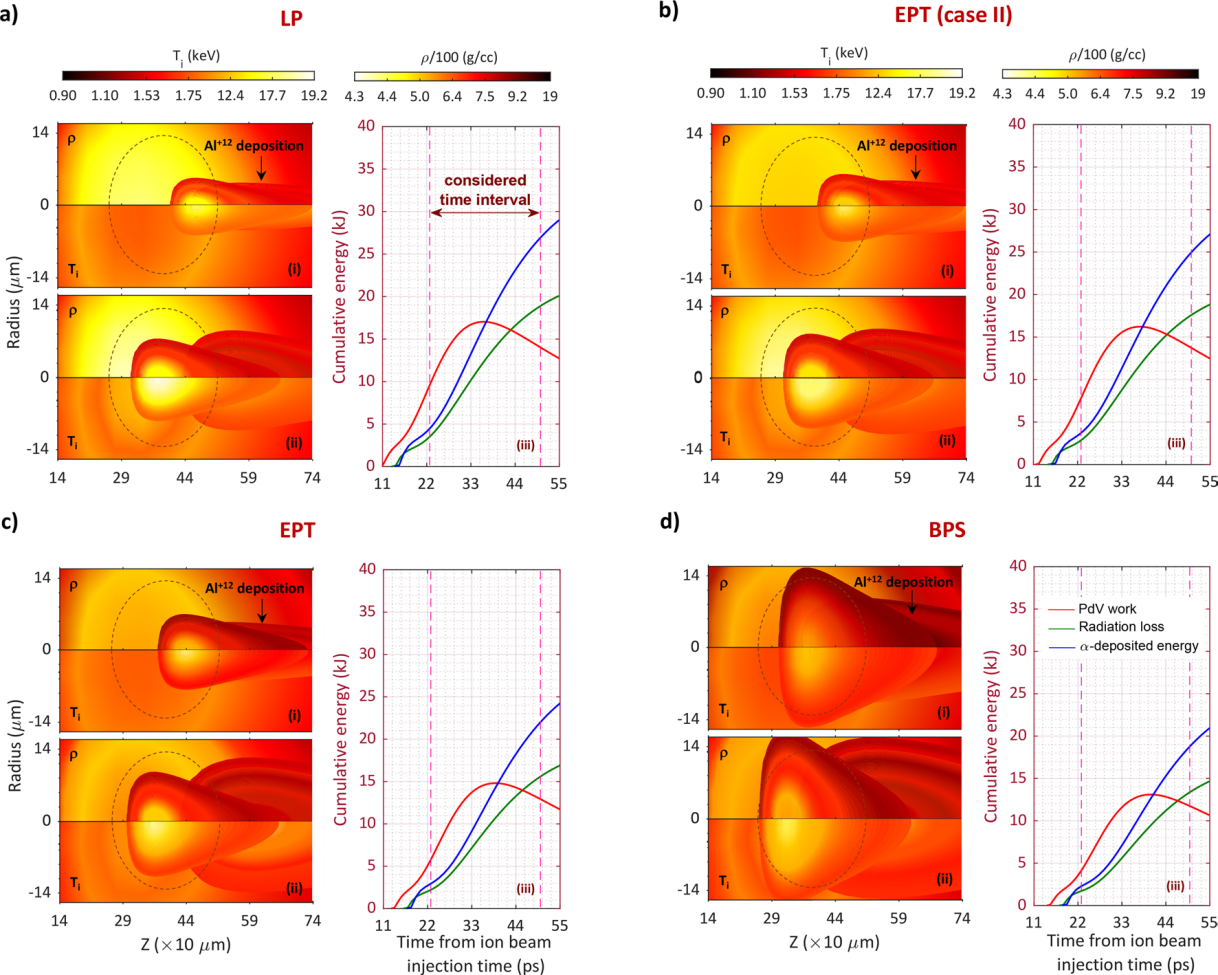
Comparison of density, ρ, and ion temperature, T_i_, maps of imploded DT target for four different stopping frameworks. (**a**) LP, (**b**) EPT (case II), (**c**) conventional EPT, and (**d**) BPS. The comparisons have been carried out for two different time values. (**i**) just after the end of the injected aluminum beam (0.023 ns), and (**ii**) when the burn wave is propagating in plasma volume (0.05 ns). To further analyses, the time evolutions of the cumulative hot-spot energies are also illustrated for different stopping formalisms (see (**iii**)). The energy curves show PdV work (red line), radiation loss (green line), and α-deposited energy (blue line). The brown elliptic curve expresses the initial position of the dense DT core.

Figure 4 shows the two-dimensional density (half-top) and ion temperature (half-down) distributions along the aluminum beam path in coronal and dense core plasma volume at the end of the pulse (23 ps), and during the beam propagation (50 ps). To better comparison, we zoned the maps into 140 μm < *z* < 740 μm and − 14.8 μm < *r* < 14.8 μm. The scale of density (normalized to 100 g/cc) is set between 430 and 1900 g/cc, while that of temperature (normalized to 1 keV) is set between 0.9 and 19.2 keV. Regardless of the stopping models employed in the simulation code, one can obtain that in all cases the aluminum beam penetrates the target and reaches the high-dense DT core after 23 ps. At this time, Al^12+^ ions deposit a large part of their energy in plasma volume, which can subsequently cause thermonuclear power production. The latter can spontaneously heat the plasma confined within Al^12+^ ions deposition area. From figure, the temperature increase in dense core is higher than coronal plasma. Furthermore, as seen in map profiles, the temperature increase is more apparent at almost the central areas of beam propagation zoned at the dense DT core limit. Perhaps the most appropriate answer to this issue will be Fig. 3aii. From this figure, the highest density distribution of Al^12+^ ions is concentrated in nearly the central limits of the accelerated beam. Thus, it can be expected that the beam-plasma interactions enhance led to a few keV increase in temperature at related plasma limits, while that of the marginal areas are limited to less temperature increase. In contrast, as can be seen, the density increases at the marginal areas of deposited beam path. In fact, as a consequence of the fast energy deposition provided by the aluminum beam, a shock wave is lunched forward into the plasma volume. The shock region is almost finite; however, it is strong enough to accumulate the dense volume limited within the shock region. Therefore, the density will increase so that from our simulation results, reaches a peak density of more than 640 g/cc for different stopping theories. The latter can be seen during the burn propagation (50 ps) as well.

According to the conservative ignition criterion $\rho_{hs} R_{hs} T_{hs} > 6g.cm^{ - 2} .keV$^61^, ignition occurs when the temperature of hot-spot reaches 10 keV. In frames corresponding to 23 ps, one can see in all stopping cases, Al^12+^ ions provide the energy required for ignition condition. However, as expected, this temperature increase in LP and EPT (case II) is more apparent compared to conventional EPT and BPS models, which exhibit an improvement in ignition condition. The key answer of this result lies in aluminum penetration depth in LP and EPT (case II) models (the so-called BP) with maximum exit stopping power values compared with conventional EPT and BPS (see Fig. 6). This conclusion is also acceptable at 50 ps.

The temporal evolutions of the cumulative hot-spot energies are also illustrated in Fig. 4. The hydrodynamic compression (PdV work; red) leads to an increment in the total hot-spot energy. This increasing trend will continue until any alpha heating (blue) and the hydrodynamic equilibrium time in IFI. The procedure is then suppressed due to hydrodynamic expansion occurred after hydrodynamic equilibrium (PdV work; red), and Bremsstrahlung radiation losses (green). Considering the curve evolutions within the considered time intervals of 23 ps ≤ t ≤ 50 ps, it can be deduced that the performance of stopping models is almost similar. Nonetheless, the LP formalism exhibits a faster rate and higher values for the cumulative energy curves compared to other stopping methods. In this comparison, our modified EPT framework (case II) has the most agreement with LP method, while that of the conventional EPT and BPS methods are in the third and fourth positions, respectively. To further study, a schematic summary of our main consequences is also given in Supporting Information, Fig. S2.

## Conclusions and overview

The work presented in this research explores the role of stopping power model in qualitative study of the ignition/burn conditions in ion fast ignition (IFI) of inertial confinement fusion (ICF) relevant plasma targets. To this aim, we employed three slowing-down methods, including Li-Petrasso (LP), Brown-Preston-Singleton (BPS), and effective potential theory (EPT). As another attempt, we also probed the modifications of collision operator to the original EPT model, and compare to other three related stopping theories. In order to modify the original EPT, we used the modification technique followed in LP framework. The latter resulted in modifying conventional EPT model accounting for the third- to fifth-order terms of collision operator. We found an improved coefficient,$1 + {2 \mathord{\left/ {\vphantom {2 {(5}}} \right. \kern-0pt} {(5}}\ln \overline{\Xi }),$ in the second-term of expansion which indicated the dependance of the second term on the generalization of Coulomb logarithm,$\ln \overline{\Xi }.$ We found that the fourth- and the fifth-order terms have almost the same effect on our modified EPT model. Thus, the orders of higher than five can be neglected for simplicity. The molecular dynamics (MD) simulations agreed well with our modified stopping framework. In addition, there was nearly a well agreement between our modified model and LP theory in weakly/moderately coupled plasmas. To study the role of different stopping models in IFI scenario, we employed the cone-in-shell configuration followed in indirect-drive IFI scheme. We assumed a laser-accelerated aluminum beam profile as the ignitor and investigated its generation by the simulations with code EPOCH^59^. The fuel scheme was chosen based on the design followed at the first stage of FIREX-I project^38^. In addition, to simulate the target, we used MULTI2D code^39,40^. The importance of the implosion in the cone tip deformation was discussed. Furthermore, taking into account the aluminum beam parameters, such as divergency, average energy, and energy spread, the core heating properties were analyzed for the considered stopping frameworks. The main results can be summarized as follows:

(i) For four considered stopping model, the simulated energy deposition of quasi-monoenergetic aluminum beam obviously clarifies that the ignitor beam does not deposit its total energy into the dense core, so that the fractional of it will be deposited along its path in the coronal plasma volume.
(ii) Comparison of minimum ignition energy values in four stopping cases expresses an agreement for imploded DT plasma at the incident beam injection time. Upon this declaration, our modified EPT model and LP have similar ignition energies so that their difference is about 1.74%. Meanwhile, compared to LP, the reported value increases to 3.86% and 7.47% for EPT and BPS, respectively.
(iii) For all stopping models, aluminum ions provide the temperature required for ignition condition (10 keV) at its considered pulse end (23 ps). However, the latter is more apparent for our modified EPT formalism and LP theory. This conclusion is also acceptable during the burn wave propagation.
(iv) From the hot-spot cumulative energies point of view, the performance of all stopping frameworks is in agreement. Nevertheless, the LP formalism indicates a faster rate and higher values compared to other stopping frameworks. Moreover, our modified EPT model has the most agreement with LP method (with a discrepancy of $\sim$ 9%), while that of the conventional EPT (with a discrepancy of $\sim$ 47% to LP) and BPS (with a discrepancy of $\sim$ 48% to LP) methods maintain the third and fourth contributions in accelerating the ignition time, respectively.

## Methods

### Charged particle stopping-power calculations

For this comparison, a fast test particle with mass *M*, charge *q*, and initial speed *V*_0_ was launched in the *x*-direction in an unmagnetized plasma. The relative mass defined as m_rel_ = *M*/*m* was considered for this research, where *m* regards as the mass of field particle in OCP. For this study, m_rel_ = 1000 expressed approximately the ions-electrons interaction (the proton to electron mass ratio is approximately m_rel_ = 1836). The relative mass of m_rel_ = 1 expressed ions interacting with ions or electrons interacting with electrons. Also, the stopping power and initial speed (*V*_0_) of fast test charge were in units of $k_{B} T/a$, and $V_{T} = \sqrt {2k_{B} T/m} ,$ respectively, where $a = (3/4\pi n)^{1/3}$ was the average inter-particle spacing in a plasma with temperature $T$, and density $n.$

### Large-scale atomic/molecular massively parallel simulator (LAMMPS)

The MD simulations were performed using LAMMPS developed by Sandia National Laboratory^37^. The cubic periodic boundary condition was used, corresponding to three different domain lengths which were defined based on the coupling strength given in Table 1. Moreover, for the unmagnetized plasma, we employed $3.1 \times 10^{4} \omega_{p}^{ - 1}$ for thermodynamic equilibrium under a velocity scaling thermostat.

**Table 1:** Simulation parameters used for calculating the molecular-dynamics (MD) stopping power.

Simulation parameters	Coupling strength (Γ)	Number of particles (N)	Domain length (L(a))	Time step (Δt (ω_p_^−1^))	The length of simulation time (ω_p_^−1^)
Considered values	0.1	5.0 × 10^4^	59.4	2.5 × 10^–3^	20
	1.0	1.0 × 10^4^	34.7	1.0 × 10^–2^	20
	10	5.0 × 10^3^	27.6	1.0 × 10^–2^	40

### Target structure and parameters for simulation

In FIREX-I, a polystyrene (CH) and cryogenic D_2_ or DT shell target will be imploded to generate a high-density core plasma^38^. We performed an analytical 2D simulation of this configuration to carry out our hypotheses in this paper.

An Au cone with an opening angle of 30° is attached to a spherical CH (6 μm thick)-DT (10 μm thick) shell contained the radius of and 250 μm. The scheme relies on filling the capsule with a low-density (10^–4^ g/cc) DT gas. This technique regards as a simple potential target species in search for naturally enhancing the ignition process in ICF targets. In fact, compared with the encapsulated CH-DT layer, the central DT gas contains much lower density, which can subsequently lead to take up more entropy during the implosion phase. Therefore, as the temperature of the central region increases, a hot-spot is formed, which itself can act as a starting point for ignition to occur.

The structure of the guide cone consists of a 10.9 μm tip inner radius, 7 μm tip thickness, and 5 μm wall thickness near the tip, which has an offset of 50 μm from the shell center. In our simulation, the target was first driven by a temporally shaped, 24-ns long laser pulse with a peak power of approximately 354 TW and a total laser energy of 1.314 MJ (see Fig. 2a). To simulate implosion process we consider 186 laser beams that are divided into two groups of 93 laser pulses, one of which is injected into the hohlraum from above laser entrance hole (LEH), whereas the other is injected from below LEH. Furthermore, the pulses are injected into the hohlraum at an angle of 50^˚^ relative to the hohlraum axis to produce X-ray radiation.

## Modelling

### Simulation technique for indirect-drive ion fast ignition (IFI)

In indirect-drive IFI simulations, we are facing two different issues. The first makes simulation of the hohlraum-cone-shell target design to analyze the implosion/burn condition with a suitable capsule. The second makes simulation of the laser-accelerated ion beam inside the attached cone to analyze the beam characteristics and propagation in IFI relevant plasma targets. The related cases made us to conduct our hypotheses by using the two-dimensional (2D) simulation code of MULTI^39,40^ to investigate the IFI of ICF relevant plasma targets in implosion and burn phases. However, in principle, the code MULTI2D is intended to simulate conventional (direct/indirect) ICF problems (ns pulses, 10^15^ W/cm^2^) using hydrodynamic description of the plasma (without kinetic effects). For IFI, it can be used to study some secondary aspects, including compression of the matter, pre-pulse heating, and etc., but not to main process of laser interactions at fs time scale, such as those we have in the target normal sheath acceleration (TNSA) or radiation pressure acceleration (RPA) concepts. The latter led us to use the two-dimensional 2D3V particle-in-cell (PIC) code (EPOCH)^59^ as the second auxiliary code of this research to investigate the acceleration and controllability of ion beam in cone-attached targets.

### MULTI2D hydrodynamic simulations

The simulation box is at the *r-z* cylindrical coordinates. The size of each cell in simulation box is the same in both directions and equals to Δ*r* = Δ*z* = 1 μm, and the time step of the simulation is Δ*t* = 3 fs. Hydrodynamic equations are solved by the Lagrangian-based method. The number of considered cells for DT target are 90 (for CH layer), 90 (for DT shell), and 60 (for DT gas) at both directions. Moreover, in all calculations, the computational grid points are 320 × 280, and most of them are cumulated on the shell and cone and around them.

### EPOCH particle-in-cell (PIC) simulations

**Figure 5:**
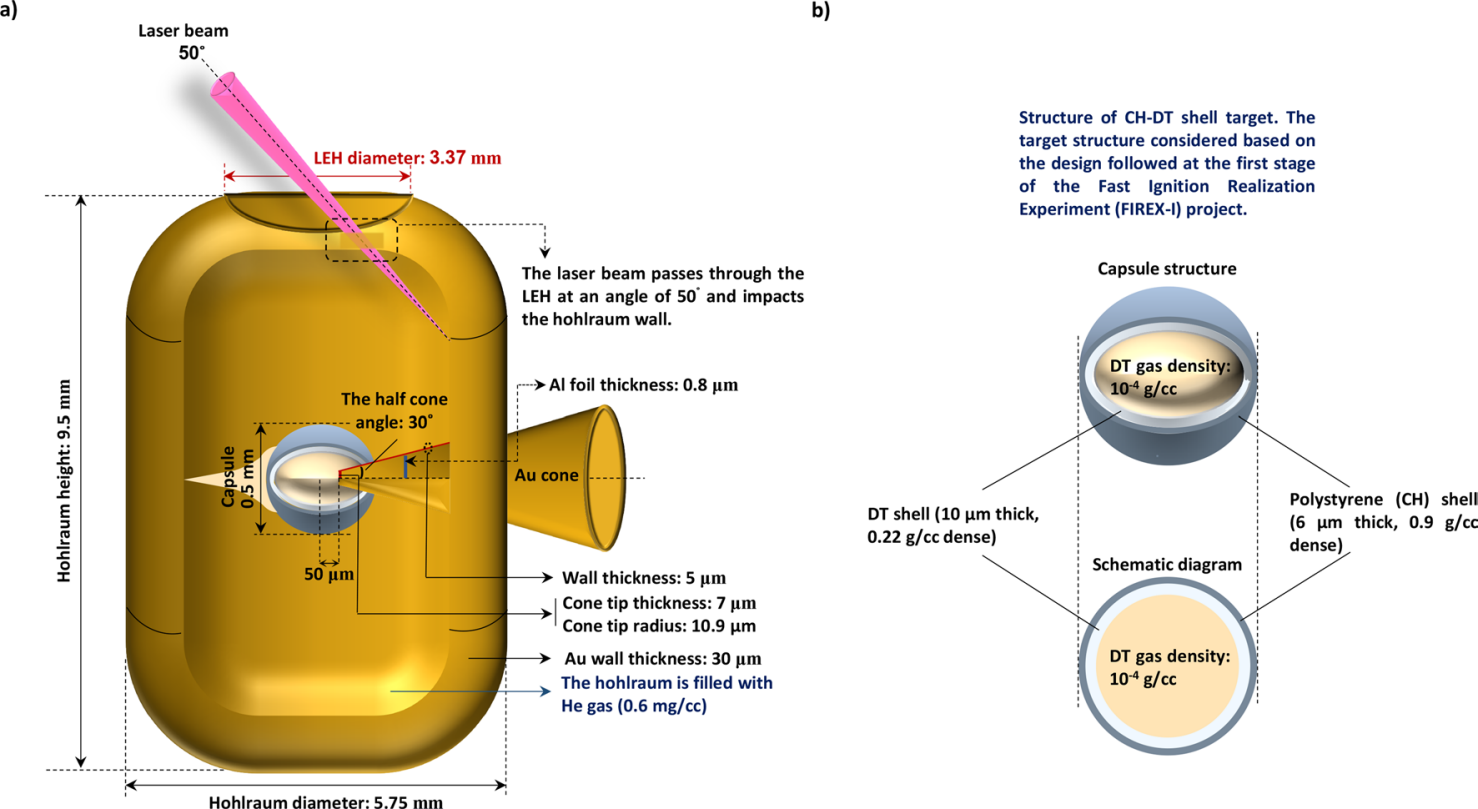
(**a**) The schematic illustration of a cone-in-shell target inside the hohlraum. The ns laser beams are injected into the hohlraum at an angle of 50^˚^ relative to the hohlraum axis to generate X-ray radiations, which can lead to the capsule implosion. At this stage, we only consider the target implosion inside the hohlraum, and ignore the ignition phase caused by the laser-foil interaction in the Au cone. (**b**) The structure of CH-DT shell target considered for simulation. The scheme was chosen based on the design followed at the first stage of FIREX-I project.

We focused on interaction of ultra-intense short laser pulses with thin aluminum foils under the effect of radiation pressure acceleration (RPA) regime. Our initial target configuration considered in code EPOCH, contains an Au cone with density of 19.3 g/cc, and a thin foil inside it, as depicted in Fig. 5. The foil is made of pure aluminum with density of 2.7 g/cc and thickness of 800 nm.

One of our challenges in simulating the acceleration of Al ions was to estimate a relatively suitable size for the simulation box. We had to adjust the size of box in such a way that the spatial distribution of the accelerated ions could almost cover the imploded core plasma close to the maximum compression region (i.e.$\sim$ 436.4 g/cc at 25.24 ns in Fig. 2ciii). Based on our simulation results illustrated in Fig. 2ciii, the maximum compression, ρ $\sim$ 436.4 g/cc, obtained at about 281 μm ≤ *z* ≤ 470 μm in cylindrical geometry, which is at least 260 μm away from cone tip. Therefore, to analyze the acceleration procedure, we adjusted the size of simulation box 770 μm × 120 μm, at the *x*–*y* plane in EPOCH framework. Under this assumption, the grid size Δ*x* = Δ*y* = λ/100, and the step size of Δt = 0.013 fs were considered, where λ = 0.8 μm is the wavelength of incident laser pulse. We assumed 42 cells per wavelength, where each cell had 27 macro-particles in total, and each species represented by 9.

Our second step was to characterize the incident laser parameters. The studies carried out in laser-ion acceleration scenario, have been shown that the circular class of polarization requires accurate adjustment of laser and irradiated target parameters, precisely for a small laser spot, and reduces the power and energy of incident pulse^44^. Therefore, the lasers produce usually the pulses in linear class of polarization^46,62^. The latter prompted us to perform our simulations benefitting from linear polarization (LP). In this regard, we assumed the *y*-direction LP pulse with the dimensionless amplitude of a_0,LP_ = 17 (peak intensity $\sim$ 1.25 × 10^21^ W/cm^2^), propagates inside the cone from right to left under the normal incidence, and is focused at the center of Al foil which is approximately 500.8 μm away from the cone tip (see Fig. 3). The pulse has Gaussian shape with the focal spot of σ = 5 and pulse duration of 30 T_0_, where T_0_ = λ/c = 2.6 fs, in full-width at half maximum (FWHM). This simulated laser pulse potentially ionizes the cone gold walls and Al foil to state charges of Au^40+^, and Al^12+^, respectively, which satisfies Bethe law^63^. Therefore, we consider a quasi-neutral plasma and ion beam consisted of Au^40+^, and Al^12+^ ions, respectively.

As the last step of simulation, we were faced a contaminated cone, since the initial target implosion had led to the presence of fuel plasma with variable densities at different spatial distances. From another perspective, the cone was surrounded by an imploded DT plasma which according to our simulation results, approximately ionized the cone wall to state charges of Au^10+^. These cases attempted us to consider the presence of DT plasma at the grids of simulation using the data obtained from Fig. 2ciii.

### Energy deposition of aluminum ions in imploded target

The aluminum beam characteristics obtained in Fig. 3 allows us to have a well-collimated and more accurate localized energy deposition in imploded core area, specifically the hot-spot volume. Nevertheless, the key attributes of interactions among Al^12+^ ions (as test particles) and an imploded core plasma are described by friction force, which regards as the average force on each individual Al^12+^ ion exerted by the rest of the imploded plasma species. These interactions create the basis of many macroscopic transport exclusivities, such as diffusion, relaxation rate, and/or conductivity, which all in turn will be effective in the imploded core ignition/burn phase. Since, the friction force is conventionally a stopping force which acts anti-parallel to the velocity of a specified test particle, different stopping formalisms maintain prominent role in expressing the hydrodynamic evolutions of core heating. In Fig. S1, we investigated the evolution of four different stopping frameworks (i.e. LP, BPS, EPT, and EPT (case II)) in plasma with different coupling strengths. In this section, we want to explore how different stopping theories can predict the core heating procedure. Note that our aim is not to choose a particular stopping model among others. Rather, our main goal in this research is to simulate and compare the hydrodynamic evolutions by benefitting from under-considered stopping frameworks.

In Fig. 3, the density distribution of the laser-accelerated Al^12+^ ions was demonstrated in detail until they inject the DT core plasma. Since, the inter-particle interactions among Al^12+^ ions and imploded plasma is irrefutable, as a first step, we explore the energy deposition of aluminum ions in DT plasma volume. In other words, by using the four under-assumption stopping models as well as examining the Bragg peak (BP) of the accelerated Al^12+^ ions, we intend to find the range of aluminum beam in imploded plasma.

**Figure 6:**
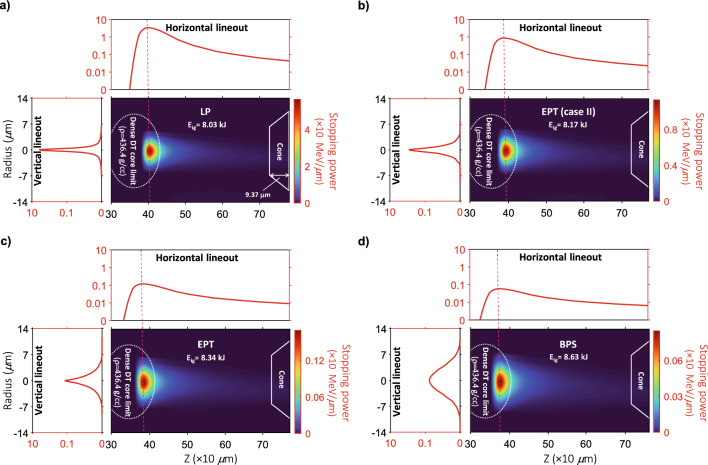
Schemes of the Al^12+^ ion beam energy density deposited in imploded DT plasma target for four different stopping power frameworks. (**a**) LP, (**b**) EPT (case II), (**c**) conventional EPT, and (**d**) BPS. The maps correspond to quasi-monoenergetic aluminum ions with the distribution energy of E_ave,Al_ = 459 keV, energy spread of ΔE_Al_/E_Al_ = 0.24, and the opening angle of *θ* $\sim$ 5.6^˚^. To more comparison, the one-dimensional horizontal and vertical distribution lineouts are plotted for each stopping model. The white elliptic and trapezoidal curves express the initial position of the dense DT core and cone, respectively.

Figure 6 presents the energy deposition of quasi-monoenergetic Al^12+^ ions profile for four stopping power models that were considered. Obviously, the maps clarify that Al^12+^ ions do not deposit their total energy into the dense core, so that the fractional of it will be deposited along their path in the coronal plasma. This result was also confirmed in references^48,64^, where the energy deposition profile of the proton beam has been investigated for the pure (reference^48^), and contaminated (reference^64^) DT plasma in IFI scenario.

As shown by the comparison, for LP stopping framework, the aluminum beam penetrates shallower in imploded plasma regime. In this case, compared with other stopping formalisms, one can observe some changes in the energy deposition profile. These changes originated from the accumulative effects of interactions among Al^12+^ ions and the background plasma particles can affect narrowing of the peak, reduction of peak height, and increasing injection energy for LP theory. In contrast, the BPS profile has the highest lateral dispersion of the beam corresponded to the lowest energy deposition, and deeper propagation in plasma volume. To interpret the latter results, perhaps our findings in Fig. S1 are the most appropriate answer (see Supporting Information). From Fig. S1, for ions-ions interactions (m_rel_ = 1), the obtained values in LP stopping power are significantly higher than those predicted by other stopping models at Γ ≤ 1. Furthermore, BPS model has the least agreement with other stopping frameworks. Since, the imploded ICF plasma is almost moderately/weakly coupled, thus, the observed behavior of BP in Fig. 6, seems normal. Moreover, from Fig. 6, our modified EPT stopping power (case II) has more agreement with LP model than the conventional EPT formalism.

To further study, we evaluated the ignition energy, E_ig_, for each case in Fig. 6. To this aim, by using the obtained data for each stopping frameworks, we first calculated the distribution power within 10.42 ps with time step 0.01. In each time step, the calculated energies were multiplied by the number of accelerated aluminum ions in that step. Ignition energy values were finally denoted as the sum of all evaluated multiple energies.

Dependently of the four considered stopping formalisms, it is remarkable that the ignition energies are in agreement for imploded DT plasma at injection time. From figure, LP and EPT (case II) have similar ignition energies so that their difference is approximately 1.74%. Meanwhile, compared to LP, the reported value increases to 3.86% and 7.47% for EPT and BPS, respectively. In other words, it seems that LP and EPT (case II), expect better beam-core coupling and subsequently, lower ignition energies than those obtained for EPT and BPS.

## Supplementary Information

Supplementary Information.

## Data Availability

All data generated or analyzed during this study are included in this research paper.
