# Supplementary material for: Simulation study of cone-in-shell target for indirect-drive ion fast ignition concept under the theory of an effective interaction potential
Source: Sci Rep. 2023 Jun 10;13:9454. doi: 10.1038/s41598-023-36597-0 (PMC10257665; doi:10.1038/s41598-023-36597-0)
Supplement: Supplementary file 1 — Supplementary Information. [file 41598_2023_36597_MOESM1_ESM.docx]

*Supporting Information for:*

Simulation study of cone-in-shell target for indirect-drive ion fast ignition concept under the theory of an effective interaction potential

Mahsa Mehrangiz[[1]](#footnote-1) and Soheil Khoshbinfar[[2]](#footnote-2)

*Department of Physics, Faculty of Sciences, University of Guilan, P.O. Box: 41335-1914, Rasht, Iran*

**Figure S1:** Comparisons of stopping power for the case of four different stopping models of LP, BPS, EPT, and case II. The analyses are followed at the coupling strength values, Γ, of 0.1, 1, and 10, and two relative masses of m_rel_=1000, and m_rel_=1. Red-solid curve refers to original EPT model; gold-dashed, and pink-dotted curves, respectively introduce the effects of case II, BPS, and LP formalisms. To further comparison, the percentage difference of each stopping curves to case II results is expressed in the bar graphs for the two relative masses.

Theories of LP and BPS compare similarly with EPT and case II here as well. However, the results clearly show that LP and BPS stopping curves show poor performance in strongly coupled regimes (Γ=10). This was also declared in reference 25. Unlike LP and BPS, EPT and case II show a well-documented treatment for all coupling strengths. As is seen, for the massive projectile (m_rel_=1000), and around the Bragg peak, the LP and case II predictions are nearly similar for Γ=0.1 and Γ=1.0, so that from figure S1a_iv_ the difference of models will be less than 10% in weak coupling (Γ=0.1). In contrast, for m_rel_=1, the obtained values in LP formalism are significantly higher than the values predicted by other stopping theories at weakly/moderately coupling. From figure, we can conclude that BPS model has the least agreement with other stopping curves, so that for m_rel_=1, and the strong coupling regime the maximum difference of BPS theory and other stopping frameworks will reach about 90%, which will be related to case II (figure S1b_iv_).

**Figure S2:** Schematic comparison of the role of four under-consideration stopping models (i.e., LP, EPT (case II), conventional EPT, and BPS) in DT core heating. **a_i_)** comparison of density and ion temperature at two different times of 23 ps (after the end of the pulse), and 50 ps (when the burn wave is propagating), **a_ii_)** contribution of stopping models to hot-spot temperature increase at 23 ps, and **b)** contribution of stopping models in accelerating the ignition time.

Taking into account the simulated results clarified in this paper, at the end of this research we have summarized the main consequences of our investigations in figure S2. According to the charts, in the early moment of core heating evolutions, all stopping models are almost in agreement. Nevertheless, over time (i.e., the burn wave propagation), the LP and BPS models lead to the fastest and slowest rates, respectively. In addition, by accepting this criterion that ignition occurs when the temperature of hot-spot reaches 10 keV, we can expect that LP and EPT (case II) express more suitable condition. Therefore, in IFI scenario, our modified EPT model (case II) shows almost a similar behavior to LP method. In other words, the only main difference between these two models can be their performance in strongly coupled plasma regimes (see figure S1).

**Modified Li-Petrasso (LP) stopping power formalism**

To investigate the role of fourth-term of the Fokker-Planck (FP)-based collision operator in Li-Petrasso (LP) stopping formalism, we first consider the distribution function as below:

$f(v,t+\Delta t)=\int f(v-\Delta v,t)F(v-\Delta v,\Delta v)d\Delta v.$ (S1)

In a plasma volume, most Coulomb collisions lead to a small change in a specified particle’s velocity. Thus, one can expect$F(v-\Delta v,\Delta v)$to be highly peaked around$\Delta v=0$in the second argument if$\Delta t$is small. Therefore, it may be appropriate to treat$\Delta v$as a small quantity and to expand$f\left(v-\Delta v,t\right)$and$F\left(v-\Delta v,\Delta v\right)$in the first argument, we would have:

$f(v,t+\Delta v)=\int[f\left(v,t\right)F\left(v,\Delta v\right)-\Delta v\frac{\partial}{\partial v}f\left(v,t\right)F\left(v,\Delta v\right)+\frac{\left(\Delta v\right)^{2}}{2!}\frac{\partial^{2}}{\partial v^{2}}f\left(v,t\right)F\left(v,\Delta v\right)$ (S2)

$$\begin{matrix} \begin{matrix} & & & \end{matrix} & & & -\frac{\left(\Delta v\right)^{3}}{3!}\frac{\partial^{3}}{\partial v^{3}}f\left(v,t\right) \end{matrix}F\left(v,\Delta v\right)+\frac{\left(\Delta v\right)^{4}}{4!}\frac{\partial^{4}}{\partial v^{4}}f\left(v,t\right)F\left(v,\Delta v\right)-...]d\Delta v.$$

As the sum of all probabilities of velocity changes is unity, for all *v* values we can use the following notations:

$\left\langle\Delta v\right\rangle=\int F(v,\Delta v)\Delta vd\Delta v,$ (S3)

$\left\langle\left(\Delta v\right)^{2}\right\rangle=\int F(v,\Delta v)\left(\Delta v\right)^{2}d\Delta v,$ (S4)

$\left\langle\left(\Delta v\right)^{3}\right\rangle=\int F(v,\Delta v)\left(\Delta v\right)^{3}d\Delta v,$ (S5)

$\left\langle\left(\Delta v\right)^{4}\right\rangle=\int F(v,\Delta v)\left(\Delta v\right)^{4}d\Delta v.$ (S6)

The rate of changes in the distribution function due to collisions can be considered as:

$C(f)=\frac{\partial f(v,t)}{\partial t}|_{collisions}=\lim_{\Delta t\to0} \frac{f\left(v,t+\Delta t\right)-f(v,t)}{\Delta t}.$ (S7)

Using equations (S3-S6) we would have:

$C(f_{b})=-\frac{\partial}{\partial v}\left(\frac{\left\langle\Delta v\right\rangle}{\Delta t}f_{b}\right)+\frac{\partial^{2}}{\partial v^{2}}\left(\frac{\left\langle\left(\Delta v\right)^{2}\right\rangle}{2\Delta t}f_{b}\right)-\frac{\partial^{3}}{\partial v^{3}}\left(\frac{\left\langle\left(\Delta v\right)^{3}\right\rangle}{6\Delta t}f_{b}\right)+\frac{\partial^{4}}{\partial v^{4}}\left(\frac{\left\langle\left(\Delta v\right)^{4}\right\rangle}{24\Delta t}f_{b}\right),$ (S8)

where *f* is in terms of velocity, *v*, and time, *t*. Benefitting from Rosenbluth’s method followed in reference 20, as the next step we elaborate on calculating the Coulomb scattering for the non-relativistic particles. To this aim, we first consider the following equations:

$\left\langle\Delta v^{\mu}\right\rangle=\sum_{b} dv'f_{b}(v')\int d\Omega\sigma(u,\Omega)\Delta v^{\mu},$ (S9)

$\begin{matrix} \left\langle\Delta v^{\mu}\Delta v^{\nu}\right\rangle=\sum_{b} dv'f_{b}(v')\int d\Omega\sigma(u,\Omega)\Delta v^{\mu}\Delta v^{\nu}, \\ \begin{matrix} & \vdots& \end{matrix} \end{matrix}$ (S10)

where μ, and ν, … correspond to the coordinate axes. $f_{b}\left(v'\right)$regards as the distribution function of a single-particle, *b* is the plasma volume, which as identified, we employed to specify that the calculations are performed for a set of particles. Furthermore,$\sigma\left(u,\Omega\right)$is the differential scattering cross-section:

$\sigma\left(u,\Omega\right)=\left(\frac{Z_{a}^{2}Z_{b}^{2}e^{4}}{4m_{ab}^{2}u^{4}}\right)\frac{1}{\sin^{4}\left(\theta/2\right)},$ (S11)

where *u* is the relative velocity, *θ* is the scattering angle, and *m*_ab_ regards as the reduced mass of two particles *a*, and *b*. We follow the calculations in the spherical coordinate system. Under this assumption, the relative velocity changes of particles can be considered as follows:

$\Delta u_{L}^{1}=-2u\sin^{2}\left(\theta/2\right),$ (S12)

(S13)

(S14)

where in equations (S12-S14), the superscripts 1-3 show the *x*, *y*, and *z* axes, respectively. Moreover, to continue the calculations, the following unit vectors are used:

$e'_{1}=\mathbf{u}/u,\begin{matrix} & e'_{2}=e'_{1}\times e'_{3},\begin{matrix} & e'_{3}=e'_{1}\times e'_{2}. \end{matrix} \end{matrix}$ (S15)

Using equation (S15), it is possible to express the velocity changes of the incident particle in terms of unit vectors as follows (note that the calculations are followed at the laboratory coordinate system):

$\Delta u^{\mu}=\left(e_{\mu}.e'_{\nu}\right)\Delta u_{L}^{\nu},$ (S16)

$\Delta u^{\mu}\Delta u^{\nu}=\left(e_{\mu}e'_{\sigma}\right)\left(e_{\nu}e'_{\omega}\right)\Delta u_{L}^{\sigma}\Delta u_{L}^{\omega}.$ (S17)

Considering the general form of $\left\{\Delta u_{L}^{\mu}\right\}=\int d\Omega\sigma\left(u,\Omega\right)u\left(\Delta u_{L}^{\mu}\right),$ it is possible to calculate$\Delta u_{L}$along the three coordinate axes:

(S18)

where$\lambda_{D}$and$b_{\min}$regard as Debye length and the minimum value of collision parameter, respectively so that$\ln(\lambda_{D}/b_{\min})$is the well-known Coulomb logarithm. For simplicity, we have assumed that the particles *a* and *b* have an equal atomic number (*Z*_a_=*Z*_b_=1). Moreover, we denote the ratio of$\lambda_{D}/b_{\min}$by the letter “*D*” in this appendix. Therefore, equation (S18) can be considered as:

$\left\{\Delta u_{L}^{1}\right\}=\left(-4\pi e^{4}/m_{ab}^{2}u^{2}\right)\ln D.$ (S19)

Also, we have:

$\left\{\Delta u_{L}^{2}\right\}=\left\{\Delta u_{L}^{3}\right\}=0.$ (S20)

Using equations (S19, S20), we can calculate the expectation values for the velocity changes as:

$\begin{matrix} \left\{\Delta u_{L}^{\mu}\Delta u_{L}^{\nu}\right\}=\int d\Omega\sigma\left(u,\mu\right)u\left(\Delta u_{L}^{\mu}\right)\left(\Delta u_{L}^{\nu}\right) \\ \begin{matrix} \begin{matrix} & \end{matrix} & & =\left(\frac{e^{4}}{m_{ab}^{2}u}\right)\int_{0}^{2\pi} \sin\theta d\theta= \end{matrix}\left(\frac{-4\pi e^{4}}{m_{ab}^{2}u}\right). \end{matrix}$ (S21)

Taking into account$\Gamma=\left(4\pi e^{4}\ln D/m_{a}^{2}\right),$equation (S21) can be written as:

$\left\{\Delta u_{L}^{\mu}\Delta u_{L}^{\nu}\right\}=\frac{-4\pi e^{4}}{m_{a}^{2}m_{b}^{2}u}\left(m_{a}+m_{b}\right)^{2}\xrightarrow{\therefore}\left\{\left(\Delta u_{L}^{1}\right)^{2}\right\}=\frac{-\Gamma}{\ln D}\frac{\left(m_{a}+m_{b}\right)^{2}}{m_{b}^{2}}.$ (S22)

In the same way we have:

(S23)

$$\xrightarrow{\therefore}\left\{\left(\Delta u_{L}^{2}\right)^{2}\right\}=\left\{\left(\Delta u_{L}^{3}\right)^{2}\right\}=\frac{\left(m_{a}+m_{b}\right)^{2}}{um_{b}^{2}}\Gamma\left(1-\frac{D^{2}}{2\ln D}\right).$$

So far, the velocity changes of the incident particle in the laboratory coordinate system were investigated. In the following, we will calculate the velocity changes in the center of mass coordinate,$\Delta v^{\mu}.$We start with equation (S24):

$\left\{\Delta v^{\mu}\right\}=V+\frac{m_{b}}{m_{a}+m_{b}}\left\{\Delta u^{\mu}\right\},$ (S24)

where *V* regards the velocity in center of mass. As previously shown,$\left\{\Delta u_{L}^{2}\right\}=\left\{\Delta u_{L}^{3}\right\}=0$(see equation (S20)). In other words, the only non-zero component is ν=1. Therefore,$\left\{\Delta v^{\mu}\right\}$will be only considered for component number one (i.e., the *x*-axis). We will have:

$\begin{matrix} \left\{\Delta v^{\mu}\right\}=\left(\frac{m_{b}}{m_{a}+m_{b}}\right)\left(e_{\mu}.e'_{1}\right)\left(\frac{-4\pi e^{4}}{m_{a}^{2}m_{b}^{2}}\right)\frac{\left(m_{a}+m_{b}\right)^{2}}{u^{2}}\ln D \\ \begin{matrix} & & & \begin{matrix} & & \therefore\left\{\Delta v^{\mu}\right\}=\frac{-m_{a}}{m_{ab}}\Gamma\frac{u^{\mu}}{u^{3}}. \end{matrix} \end{matrix} \end{matrix}$ (S25)

$\left\{\Delta v^{\mu}\Delta v^{\nu}\right\}=\left(\frac{m_{b}}{m_{a}+m_{b}}\right)^{2}\left(e_{\mu}.e'_{1}\right)\left(e_{\nu}.e'_{1}\right)\left(\frac{-\Gamma}{\ln D}\frac{\left(m_{a}+m_{b}\right)^{2}}{m_{b}^{2}u}\right)+$ (S26)

$$\begin{matrix} \begin{matrix} & & & \end{matrix}\left(\frac{m_{b}}{m_{a}+m_{b}}\right)^{2}\left(e_{\sigma}.e'_{2}\right)\left(e_{\omega}.e'_{2}\right)\left(\frac{\left(m_{a}+m_{b}\right)^{2}}{m_{b}^{2}u}\Gamma\left(1-\frac{D^{2}}{\ln D}\right)\right)+ \\ \begin{matrix} & & & \left(\frac{m_{b}}{m_{a}+m_{b}}\right)^{2}\left(e_{\gamma}.e'_{3}\right)\left(e_{\gamma}.e'_{3}\right)\left(\frac{\left(m_{a}+m_{b}\right)^{2}}{m_{b}^{2}u}\Gamma\left(1-\frac{D^{2}}{\ln D}\right)\right) \end{matrix} \\ \begin{matrix} & & = & \left(\frac{-u^{\mu}u^{\nu}}{u^{2}}\right)\left[\frac{\Gamma}{\ln D}\frac{m_{a}+m_{b}}{m_{b}}\right]+ \end{matrix}\underbrace{\left(e_{\nu}\left(e_{3}\times e_{1}\right)\right)\left(e_{\mu}\left(e_{3}\times e_{1}\right)\right)\frac{\left(m_{a}+m_{b}\right)}{m_{b}u}\Gamma\left(1-\frac{D^{2}}{\ln D}\right)}_{(**)}, \end{matrix}$$

where taking into account equation (S27), and using (**), we can simplify equation (S26) as equation (S28):

(S27)

$\begin{matrix} \left\{\Delta v^{\mu}\Delta v^{\nu}\right\}=\frac{u^{\mu}u^{\nu}}{u^{2}}\left[-\frac{\Gamma}{\ln D}\left(\frac{m_{a}+m_{b}}{m_{b}}\right)\right]+2\Gamma\left(\frac{u^{\mu}u^{\nu}}{u^{2}}-\delta_{\mu\nu}\right)\left(\frac{m_{a}+m_{b}}{um_{b}}\right)\left(1-\frac{D^{2}}{\ln D}\right) \\ \begin{matrix} & & & = \end{matrix}\left(\frac{m_{a}+m_{b}}{m_{b}}\right)\left[-\frac{u^{\mu}u^{\nu}}{u^{2}}\frac{\Gamma}{\ln D}+\frac{2u^{\mu}u^{\nu}}{u^{2}}\Gamma\left(1-\frac{D^{2}}{\ln D}\right)-2\delta_{\mu\nu}\Gamma\left(1-\frac{D^{2}}{2\ln D}\right)\right] \\ \begin{matrix} & & & =-2\Gamma\left(\delta_{\mu\nu}-\frac{u^{\mu}u^{\nu}}{u^{2}}\right) \end{matrix}+\frac{\Gamma}{\ln D}\left(\delta_{\mu\nu}D^{2}-\frac{u^{\mu}u^{\nu}}{u^{2}}D^{2}-\frac{u^{\mu}u^{\nu}}{u^{2}}\right) \\ \begin{matrix} & & & =-2\Gamma\left(\delta_{\mu\nu}-\frac{u^{\mu}u^{\nu}}{u^{2}}\right) \end{matrix}+\frac{\Gamma}{\ln D}\left(D^{2}\left(\delta_{\mu\nu}-\frac{u^{\mu}u^{\nu}}{u^{2}}\right)-\frac{u^{\mu}u^{\nu}}{u^{2}}\right). \end{matrix}$ (S28)

Also, for the third component of velocity changes we can follow the same manner as:

$\begin{matrix} \left\{\left(\Delta u_{L}^{1}\right)^{3}\right\}=\int d\Omega\sigma\left(u,\Omega\right)u\left(\Delta u_{L}^{1}\right)^{3} \\ \begin{matrix} & & & = \end{matrix}\left(\frac{-16\pi e^{4}}{m_{ab}^{2}}\right)\int\sin^{2}\left(\theta/2\right)\sin\left(\theta/2\right)\cos\left(\theta/2\right)d\theta\\ \begin{matrix} & & & =\frac{-4\pi e^{4}(m_{a}+m_{b})^{2}}{m_{a}^{2}m_{b}^{2}}D^{4}=-\frac{\Gamma(m_{a}+m_{b})^{2}}{m_{b}^{2}\ln D} \end{matrix}D^{4}. \end{matrix}$ (S29)

Similarly, for$\left\{\left(\Delta u_{L}^{2}\right)^{3}\right\}$we have:

(S30)

Also, to calculate the velocity changes in the center of mass coordinate, we have:

$\begin{matrix} \left\{\Delta v^{\mu}\Delta v^{\nu}\Delta v^{\kappa}\right\}=\left(\frac{m_{b}}{m_{a}+m_{b}}\right)^{3}\left(e_{\mu}.e'_{\sigma}\right)\left(e_{\nu}.e'_{\omega}\right)\left(e_{\kappa}.e'_{\gamma}\right)\Delta u_{L}^{\sigma}\Delta u_{L}^{\omega}\Delta u_{L}^{\gamma} \\ \begin{matrix} \begin{matrix} & \end{matrix} & & & = \end{matrix}\left(\frac{m_{b}}{m_{a}+m_{b}}\right)^{3}\left(e_{\mu}.e'_{1}\right)\left(e_{\nu}.e'_{1}\right)\left(e_{\kappa}.e'_{1}\right)\left\{\left(\Delta u_{L}^{1}\right)^{3}\right\} \\ \begin{matrix} \begin{matrix} & \end{matrix} & & & =\left(\frac{u^{\mu}}{u}.\frac{u^{\nu}}{u}.\frac{u^{\kappa}}{u}\right) \end{matrix}\left[-\frac{\Gamma}{\ln D}D^{4}\right] \\ \begin{matrix} & & & \begin{matrix} & = \end{matrix} \end{matrix}\left(\frac{-m_{b}}{m_{a}+m_{b}}\right)\frac{\Gamma}{\ln D}\left[\frac{u^{\mu}u^{\nu}}{u^{2}}.\frac{u^{\kappa}}{u}D^{4}\right]. \end{matrix}$ (S31)

To express the expectation values of velocity components we calculated and considered equations (S32-S34):

$$\left\langle\Delta v^{\mu}\right\rangle=\sum_{b} f_{b}\left(v'\right)dv'\left\{\Delta v^{\mu}\right\}=\sum_{b} \int dv'f_{b}\left(v'\right)\left(\frac{-m_{a}}{m_{ab}}\right)\Gamma\left(\frac{\partial}{\partial v^{\mu}}\left(\frac{1}{u}\right)\right)$$

(S32)

(A33)

(S34)

Now, we elaborate on calculating the fourth-order term of velocity changes as follows:

(S35)

Also, for$\left\{\left(\Delta u_{L}^{2}\right)^{4}\right\}$and$\left\{\left(\Delta u_{L}^{3}\right)^{4}\right\}$the following steps are taken:

(S36)

As in previous calculations, for the velocity changes in the center of mass coordinate, we have:

$\begin{matrix} \left\{\Delta v^{\mu}\Delta v^{\nu}\Delta v^{\gamma}\Delta v^{\pi}\right\}=\underbrace{\left(\frac{m_{b}}{m_{a}+m_{b}}\right)^{4}\left(e_{\mu}.e'_{1}\right)\left(e_{\nu}.e'_{1}\right)\left(e_{\gamma}.e'_{1}\right)\left(e_{\pi}.e'_{1}\right)\left\{\left(\Delta u_{L}^{1}\right)^{4}\right\}}_{(I)}+ \\ \begin{matrix} \begin{matrix} & & \end{matrix} & & & \begin{matrix} & & \end{matrix} \end{matrix}\underbrace{\left(\frac{m_{b}}{m_{a}+m_{b}}\right)^{4}\left(e_{\mu}.e'_{2}\right)\left(e_{\nu}.e'_{2}\right)\left(e_{\gamma}.e'_{2}\right)\left(e_{\pi}.e'_{2}\right)\left\{\left(\Delta u_{L}^{2}\right)^{4}\right\}}_{(II)}+ \end{matrix}$ (S37)

$$\begin{matrix} \begin{matrix} \begin{matrix} & & \end{matrix} & & & \end{matrix} & & & \underbrace{\left(\frac{m_{b}}{m_{a}+m_{b}}\right)^{4}\left(e_{\mu}.e'_{3}\right)\left(e_{\nu}.e'_{3}\right)\left(e_{\gamma}.e'_{3}\right)\left(e_{\pi}.e'_{3}\right)\left\{\left(\Delta u_{L}^{3}\right)^{4}\right\}}_{(III)} \end{matrix},$$

(S38)

Substituting equation (S38) in (S37), the final result will be presented as equation (S39):

$\left\{\Delta v^{\mu}\Delta v^{\nu}\Delta v^{\gamma}\Delta v^{\pi}\right\}=\left(\frac{m_{b}}{m_{a}+m_{b}}\right)^{2}\frac{\Gamma u}{\ln D}[\frac{4D^{6}}{3u^{2}}\delta_{\mu\nu}.\delta_{\gamma\pi}+\frac{D^{2}}{2}\left(\frac{\partial^{2}u}{\partial v^{\mu}\partial v^{\nu}}.\frac{\partial^{2}u}{\partial v^{\pi}\partial v^{\gamma}}\right),$ (S39)

$$\begin{matrix} \begin{matrix} & \therefore& \left\langle\Delta v^{\mu}\Delta v^{\nu}\Delta v^{\gamma}\Delta v^{\pi}\right\rangle= \end{matrix}\left(\frac{m_{b}}{m_{a}+m_{b}}\right)^{2}\frac{\Gamma D^{2}}{2\ln D}\left(1-D^{2}+\frac{D^{4}}{3}\right)[\frac{4D^{6}}{3}\left(\delta_{\mu\nu}.\delta_{\gamma\pi}.H\right)+ \\ \begin{matrix} \begin{matrix} \begin{matrix} \begin{matrix} \begin{matrix} \begin{matrix} & & & \end{matrix} & & & \end{matrix} & & & \end{matrix} & & & \end{matrix} & & & \end{matrix} & & & \end{matrix}3\left(\frac{\partial^{2}G}{\partial v^{\mu}\partial v^{\nu}}.\frac{\partial^{2}\mathbf{\Phi}}{\partial v^{\gamma}\partial v^{\pi}}\right)], \end{matrix}$$

where according to reference 8, the vector potential$\mathbf{\Phi}$has the following useful properties:

$\begin{matrix} \begin{matrix} & \end{matrix}\nabla_{\mathbf{v}}^{2}\nabla_{\mathbf{v}}^{2}\nabla_{\mathbf{v}}.\mathbf{\Phi}(\mathbf{v})=f_{b}(\mathbf{v}), \\ \begin{matrix} & & \end{matrix}\nabla_{\mathbf{v}}^{2}\nabla_{\mathbf{v}}^{2}.\mathbf{\Phi}(\mathbf{v})=0, \\ \nabla_{\mathbf{v}}^{2}\nabla_{\mathbf{v}}.\mathbf{\Phi}(\mathbf{v})=\nabla_{\mathbf{v}}^{2}.G(\mathbf{v})=H(\mathbf{v}). \end{matrix}$ (S40)

In order to calculate the stopping power, we must first validate our obtained equations. Our comparison criterion is based on references 8 and 9. To this aim, at the first step, we will skip the fourth-order term of Taylor expansion. Using the introduced collision operator in equation (S8), C(*f*), we have:

$C\left(f_{b}\right)=-\frac{\partial}{\partial v^{\mu}}\left(f_{b}\left\langle\Delta v^{\mu}\right\rangle\right)+\frac{1}{2}\frac{\partial^{2}}{\partial v^{\mu}\partial v^{\nu}}\left(f_{b}\left\langle\Delta v^{\mu}\Delta v^{\nu}\right\rangle\right)-\frac{1}{6}\frac{\partial^{3}}{\partial v^{\mu}\partial v^{\nu}\partial v^{\kappa}}\left(f_{b}\left\langle\Delta v^{\mu}\Delta v^{\nu}\Delta v^{\kappa}\right\rangle\right),$ (A41)

$C\left(f_{b}\right)=\underbrace{-\frac{\partial}{\partial v^{\mu}}\left(f_{b}\left[-\Gamma\left(\frac{m_{a}+m_{b}}{m_{b}}\right)\frac{\partial H}{\partial v^{\mu}}\right]\right)}_{(I)}+$ (S42)

$$\begin{matrix} \underbrace{\frac{1}{2}\frac{\partial^{2}}{\partial v^{\mu}\partial v^{\nu}}\left(f_{b}\left[-2\Gamma\frac{\partial^{2}}{\partial v^{\mu}\partial v^{\nu}}G+\frac{\Gamma}{\ln D}\left(3\frac{\partial^{2}}{\partial v^{\mu}\partial v^{\nu}}G-\delta_{\mu\nu}H\right)\right]\right)}_{(II)}- \\ \begin{matrix} \begin{matrix} \begin{matrix} & \end{matrix} & & \end{matrix} & \end{matrix}\underbrace{\frac{1}{6}\frac{\partial^{3}}{\partial v^{\mu}\partial v^{\nu}\partial v^{\kappa}}\left(f_{b}\left[4\left(\frac{m_{b}}{m_{a}+m_{b}}\right)\frac{\Gamma}{\ln D}\left(\frac{\partial^{2}}{\partial v^{\mu}\partial v^{\nu}}\mathbf{\Phi}\right)\right]\right)}_{(III)}, \end{matrix}$$

(S43)

Taking into account:

and *f*_b_ as:

$$f_{b}=\left(\frac{n}{(2\pi m_{b}T_{b})^{3/2}}\right)\exp\left[-\left(v_{a}/v_{b}\right)^{2}\right],\begin{matrix} \begin{matrix} & \end{matrix}where: & T_{b}\equiv\begin{matrix} Boltzmann & temperature \end{matrix} \end{matrix}$$

we can simplify C(*f_b_*) in terms of “G” as equation below (note that here, C(*f_b_*) is considered for the ion-electron collisions. Under this assumption, we can consider$(m_{a}+m_{b})/m_{b}\equiv m_{b}/m_{a}$due to the higher charge to mass ratio of ions):

$C\left(f_{b}\right)=\Gamma\frac{\partial}{\partial v^{\mu}}\left(-\frac{\partial f_{b}}{\partial v^{\nu}}.\frac{\partial^{2}G}{\partial v^{\mu}\partial v^{\nu}}+\frac{3}{2\ln D}\frac{\partial^{3}G}{\partial v^{\mu}\partial v^{\nu}\partial v^{\nu}}-\frac{\partial^{3}G}{\partial v^{\mu}\partial v^{\nu}\partial v^{\nu}}f_{b}\right)$ (S44)

$$\begin{matrix} \begin{matrix} & & =\frac{n\Gamma}{8\pi} \end{matrix}.\frac{\partial}{\partial v^{\mu}}\left(\frac{\partial^{2}v}{\partial v^{\mu}\partial v^{\nu}}\frac{\partial f_{b}}{\partial v^{\nu}}-\frac{\partial^{2}}{\partial v^{\mu}\partial v^{\nu}}\left(\frac{v^{\mu}v^{\nu}}{v}\right)\frac{\partial f_{b}}{\partial v^{\nu}}\right)+\Gamma\frac{\partial}{\partial v^{\mu}}[-\frac{n}{8\pi}\frac{\partial^{3}v}{\partial v^{\mu}\partial v^{\nu}\partial v^{\nu}}\left(\frac{3}{2\ln D}\right)+ \\ \begin{matrix} & \begin{matrix} \begin{matrix} & & \end{matrix} & & \end{matrix} & \frac{n}{8\pi}\frac{\partial^{3}}{\partial v^{\mu}\partial v^{\nu}\partial v^{\nu}}\left(\frac{v^{\mu}v^{\nu}}{v}\right) \end{matrix}+\frac{n}{8\pi}\frac{\partial^{3}v}{\partial v^{\mu}\partial v^{\nu}\partial v^{\nu}}f_{b}-\frac{n}{8\pi}\frac{\partial^{3}v}{\partial v^{\mu}\partial v^{\nu}\partial v^{\nu}}\left(\frac{v^{\mu}v^{\nu}}{v}\right)] \\ \begin{matrix} & & = \end{matrix}\frac{n\Gamma}{8\pi}.\frac{\partial}{\partial v^{\mu}}[\frac{\partial^{2}v}{\partial v^{\mu}\partial v^{\nu}}\frac{\partial f_{b}}{\partial v^{\nu}}-\frac{\partial^{2}}{\partial v^{\mu}\partial v^{\nu}}\left(\frac{v^{\mu}v^{\nu}}{v}\right)\frac{\partial f_{b}}{\partial v^{\nu}}-\frac{3}{2\ln D}\frac{\partial^{3}v}{\partial v^{\mu}\partial v^{\nu}\partial v^{\nu}}+\frac{\partial^{3}v}{\partial v^{\mu}\partial v^{\nu}\partial v^{\nu}}\left(\frac{v^{\mu}v^{\nu}}{v}\right)+ \\ \begin{matrix} \begin{matrix} & & & \end{matrix} & & \begin{matrix} \begin{matrix} \begin{matrix} & & & \end{matrix} & & & \end{matrix} & & & \end{matrix} & \left(\frac{\partial^{3}v}{\partial v^{\mu}\partial v^{\nu}\partial v^{\nu}}f_{b}\right) \end{matrix}-\frac{\partial^{3}v}{\partial v^{\mu}\partial v^{\nu}\partial v^{\nu}}\left(\frac{v^{\mu}v^{\nu}}{v}\right)]. \end{matrix}$$

To solve equation (S45), one can consider the following simplifications:

$$v_{\mu\nu}=\frac{\partial^{2}v}{\partial v^{\mu}\partial v^{\nu}}=\frac{1}{v}\delta_{\mu\nu}-v^{\mu}v^{\nu}\begin{matrix} & and & v_{\mu\nu\kappa}=\frac{v^{\nu}}{v}\delta_{\mu\kappa}-\frac{v^{\mu}v^{\nu}v^{\kappa}}{v^{3}}+ \end{matrix}\frac{v^{\mu}}{v}\delta_{\nu\kappa}+\frac{v^{\kappa}}{v}\delta_{\mu\nu}.$$

$\therefore C\left(f_{b}\right)=\frac{4\pi e^{4}n}{2m_{a}^{2}}[\left(1-\frac{5}{6\ln D}\right)\frac{\partial f}{\partial v^{\nu}}v^{\mu\nu}+\frac{1}{\ln D}(\frac{4}{3}\frac{v^{\mu}}{v^{3}}+\frac{\delta_{\mu\nu}}{v}\frac{\partial}{\partial v^{\nu}}+\frac{v_{\mu\nu\kappa}}{6}\frac{\partial^{2}}{\partial v^{\nu}\partial v^{\mu}})].$ (S45)

In the rest of calculations, we should solve “H”, “G”, and “$\mathbf{\Phi}$” potentials by using the introduced Boltzmann function. The stopping power formalism will be calculated by substituting the obtained potentials in the expectation values of velocity:

(S46)

To continue, we use the so-called Chandrasekhar function,$\psi\left(x\right):$

(S47)

Using equation (S47), equation (S46) can be simplified as follows:

(S48)

Using:

$\frac{\partial H}{\partial v}=\frac{1}{2\underbrace{\left(v_{a}^{2}/v_{b}^{2}\right)}_{x}}.\left(\frac{nm_{a}^{2}}{T_{b}}.\frac{m_{b}}{m_{a}+m_{b}}\right)\underbrace{\left[erf\left(x\right)-\frac{2}{\sqrt{\pi}}\sqrt{x}\exp\left(-x\right)\right]}_{\mu\left(x\right)},$ (S49)

$$\therefore\frac{\partial H}{\partial v}=\frac{1}{2x}\left(\frac{nm_{a}^{2}}{T_{b}}.\frac{m_{b}}{m_{a}+m_{b}}\right)\mu\left(x\right).$$

One can also derive:

$\frac{\partial^{2}G}{\partial v^{\mu}\partial v^{\nu}}=\left(\frac{m_{b}}{m_{a}}\right)\frac{nm_{a}^{2}}{T_{b}}.\frac{1}{2x}\frac{d\mu\left(x\right)}{dx},$ (S50)

$\frac{\partial^{2}\mathbf{\Phi}}{\partial v^{\mu}\partial v^{\nu}}=\frac{nm_{a}^{2}}{T_{b}}.\frac{1}{2x}\left(\frac{m_{a}+m_{b}}{m_{b}}\right).$ (S51)

Using equations (S49-S51), we can re-written equations (S32-S34) in the general form of equations (S52-S54):

$\left\langle\Delta v^{\mu}\right\rangle=\left(\frac{4\pi Z_{a}^{2}Z_{b}^{2}ne^{4}}{2E_{a}}\right)\frac{m_{a}}{m_{b}}\ln D\mu\left(x\right)\begin{matrix} & \begin{matrix} where: & E_{a}=m_{a}/2v_{a}^{2} \end{matrix} \end{matrix},$ (S52)

$\left\langle\Delta v^{\mu}\Delta v^{\nu}\right\rangle=-\frac{4\pi Z_{a}^{2}Z_{b}^{2}e^{4}n}{2m_{a}^{2}E_{a}}\left(\frac{m_{b}}{m_{a}}\right)[\frac{d\mu\left(x\right)}{dx}-\frac{1}{\ln D}\left(\frac{d\mu\left(x\right)}{dx}+\mu\left(x\right)\right)]\ln D,$ (S53)

$\left\langle\Delta v^{\mu}\Delta v^{\nu}\Delta v^{\kappa}\right\rangle=\frac{4\pi Z_{a}^{2}Z_{b}^{2}e^{4}n}{2m_{a}^{2}E_{a}}\underbrace{\Theta\left(x\right)}_{\begin{matrix} step & function \end{matrix}}\ln\left(1.123\sqrt{x}\right).$ (S54)

Taking into account equations (S52-S54), one can calculate the conventional LP formalism as:

$\left(\frac{dE_{a}}{dx}\right)_{collision}\cong\frac{m_{a}}{2}\mathbf{v}_{a}(\sum_{n'=1}^{3} \frac{1}{\mathbf{v}_{a}^{n'-1}}\frac{\left\langle\left(\Delta\mathbf{v}\right)^{n'}\right\rangle}{\Delta x}).$ (S55)

$$\begin{matrix} \left(\frac{dE_{a}}{dx}\right)_{collision}=\frac{4\pi Z_{a}^{2}Z_{b}^{2}e^{4}n}{2E_{a}}\left(\frac{m_{a}}{m_{b}}\right)\{\{\mu\left(x\right)-\left(\frac{m_{b}}{m_{a}}\right)[\frac{d\mu\left(x\right)}{dx}- \\ \begin{matrix} \begin{matrix} \begin{matrix} & & \end{matrix} & & & \end{matrix} & & & \end{matrix}\frac{1}{\ln D}\left(\frac{d\mu\left(x\right)}{dx}+\mu\left(x\right)\right)]\}\ln D+\Theta\ln\left(1.123\sqrt{x}\right)\}. \end{matrix}$$

To modify LP formalism, we used equation (S37) and added to equation (S56):

$\left\langle\Delta v^{\mu}\Delta v^{\nu}\Delta v^{\gamma}\Delta v^{\pi}\right\rangle=\left(\frac{m_{b}}{m_{a}+m_{b}}\right)^{2}\frac{\Gamma}{\ln D}\left[\frac{20}{3}\left(\delta_{\mu\nu}\delta_{\gamma\pi}H\right)+\left(\frac{\partial^{2}G}{\partial v^{\mu}\partial v^{\nu}}.\frac{\partial^{2}\mathbf{\Phi}}{\partial v^{\gamma}\partial v^{\pi}}\right)\right]$ (S56)

$$\begin{matrix} \begin{matrix} \begin{matrix} \begin{matrix} & \end{matrix} & & \end{matrix} & & = \end{matrix}\left(\frac{m_{b}}{m_{a}+m_{b}}\right)^{2}\frac{\Gamma}{\ln D}[-\frac{\mu\left(x\right)}{2x}\left(\frac{nm_{b}m_{a}}{T_{b}}\right)+\left[\left(\frac{nm_{b}m_{a}}{2xT_{b}}\right)\frac{d\mu\left(x\right)}{dx}\right] \\ \begin{matrix} \begin{matrix} & & \end{matrix} & & & \begin{matrix} \begin{matrix} \begin{matrix} \begin{matrix} & & \end{matrix} & & \end{matrix} & & \end{matrix} & \left\{\frac{nm_{a}^{2}}{2xT_{b}m_{b}}\left(m_{a}+m_{b}\right)\Theta\left(x\right)\ln\left(\sqrt{1.427x}\right)\right\}] \end{matrix} \end{matrix}, \end{matrix}$$

$\begin{matrix} \left\langle\Delta v^{\mu}\Delta v^{\nu}\Delta v^{\gamma}\Delta v^{\pi}\right\rangle=\frac{4\pi Z_{a}^{2}Z_{b}^{2}e^{4}n}{2E_{a}}\frac{m_{a}}{m_{b}}\left(\frac{m_{b}}{m_{a}+m_{b}}\right)^{2}\{\frac{m_{b}}{m_{a}}(-\mu\left(x\right)+ \\ \begin{matrix} \begin{matrix} & & & \begin{matrix} & & & \end{matrix} \end{matrix} & & & \end{matrix}\frac{d\mu\left(x\right)}{dx}\left(\frac{m_{a}+m_{b}}{m_{b}}\right)\Theta\left(x\right)\ln\left(\sqrt{1.427x}\right))\}. \end{matrix}$ (S57)

Taking into account equations (S56, S57), one can calculate the modified LP formalism as:

$\left(\frac{dE_{a}}{dx}\right)_{collision}\cong\frac{m_{a}}{2}\mathbf{v}_{a}(\sum_{n'=1}^{4} \frac{1}{\mathbf{v}_{a}^{n'-1}}\frac{\left\langle\left(\Delta\mathbf{v}\right)^{n'}\right\rangle}{\Delta x}).$ (S58)

$$\begin{matrix} \left(\frac{dE_{a}}{dx}\right)_{collision}=\frac{4\pi Z_{a}^{2}Z_{b}^{2}e^{4}n}{2E_{a}}\left(\frac{m_{a}}{m_{b}}\right)\{(\mu\left(x\right)-\left(\frac{m_{b}}{m_{a}}\right)[\frac{d\mu\left(x\right)}{dx}-\frac{1}{\ln D} \\ \begin{matrix} \begin{matrix} \begin{matrix} \begin{matrix} & \end{matrix} & & & \end{matrix} & & & \end{matrix} & & & \left(\frac{d\mu\left(x\right)}{dx}+\mu\left(x\right)\right)])\ln D+\Theta\left(x\right)\ln\left(\sqrt{1.427x}\right) \end{matrix}+ \\ \begin{matrix} \begin{matrix} \begin{matrix} \begin{matrix} & \end{matrix} & & & \end{matrix} & & & \end{matrix} & & & \left(\frac{m_{b}}{m_{a}+m_{b}}\right)^{2}[\left(\frac{m_{b}}{m_{a}}\right)(- \end{matrix}\mu\left(x\right)+ \\ \begin{matrix} \begin{matrix} \begin{matrix} \begin{matrix} & & & \end{matrix} & & & \end{matrix} & & \end{matrix} & & & \end{matrix}\frac{d\mu\left(x\right)}{dx}\left(\frac{m_{a}+m_{b}}{m_{b}}\right)\Theta\left(x\right)\ln\left(\sqrt{1.427x}\right))]\}. \end{matrix}$$

**Figure S3:** Comparisons of the second-to-fourth-order terms of Taylor’s expansion effects on the LP stopping framework. The comparisons are expressed for m_rel_=1000, and m_rel_=1 at the coupling strength value, Γ, of 1. purple-solid curve refers to second-order; brown-dashed, and green-dash-dotted, respectively introduce the effects of third to fourth orders of FP method to the original LP formalism. To further comparison, the peak stopping power values, and the percentage difference of third and fourth stopping curves to second stopping curve are exhibited in the bar graphs for the two relative masses. Note that the second and third terms relate to the conventional LP formalism, while that of the fourth-term shows the modified form of LP stopping formula obtained in this research. Moreover, as discussed in paper, a is the average inter-particle spacing in a plasma with temperature T.

Using equations (S1-S58), we exhibited the comparisons of the original LP stopping power to our modified LP formalism (included the fourth-order-term of expansion) for m_rel_=1000 and m_rel_=1 in figure S3. As previously discussed in this paper, based on our measurements, the stopping curves are in well-agreement. However, the addition of the fourth-order term raises the ionic and electronic stopping power by approximately 4.8% and 1.2%, respectively. Thus, one can expect the cumulative effects of higher-terms on stopping power are negligible.

**Modified effective potential theory (EPT) stopping power formalism**

To derive a new expression for the conventional EPT stopping power, in the first step, we consider an equilibrium unmagnetized plasma, where the particle velocity describes by the Maxwellian distribution function, *f_s_*. Assuming$\mathbf{v}_{s}$, Z_s_, m_s_, and$\mathbf{r}_{s}$ as the velocity, atomic number, mass, and position of a particle species, respectively, one can obtain the equation of motion as:

$m_{s}\frac{d\mathbf{v}_{s}(t)}{dt}=Z_{s}e\mathbf{E}_{fl}(\mathbf{r}_{s}(t),t),$ (S59)

which has the solution:

$\mathbf{v}_{sf}(t_{f})=\mathbf{v}_{si}(t_{i})+\frac{Z_{s}e}{m_{s}}\int_{t_{i}}^{t_{f}} \mathbf{E}_{fl}(\mathbf{r}_{s}(t'),t')dt',$ (S60)

where$\mathbf{E}_{fl}$ shows the fluctuating electric field of a discreteness plasma particle species. For the case of a moving particle into a plasma, the value of the fluctuating field changes randomly. Therefore, one cannot expect to obtain an exact value$\mathbf{v}_{sf}(t)$. The process can be defined by introducing a transition function,$f'_{s}\left(\mathbf{v}_{sf},t_{f}|\mathbf{v}_{si},t_{i}\right)$, as the probability that the particles velocity is$\mathbf{v}_{sf}$at the final time$t_{f}$when it is$\mathbf{v}_{si}$ at the initial time of$t_{i}$. In this case, it expects that the distribution function at$t_{i}+\Delta t$complies with equation (S61) at *t_i_* through:

$f_{s}(\mathbf{v}_{sf},t_{i}+\Delta t)=\int f_{s}(\mathbf{v}_{si},t_{i})f'_{s}(\mathbf{v}_{sf},t_{i}+\Delta t|\mathbf{v}_{si},t_{i})d^{3}\mathbf{v}_{si},$ (S61)

where substituting$\Delta\mathbf{v}_{s}=\mathbf{v}_{sf}-\mathbf{v}_{si}$, equation (S61) satisfies the relation:

$f_{s}(\mathbf{v}_{sf},t_{i}+\Delta t)=\int f_{s}(\mathbf{v}_{sf}-\Delta\mathbf{v}_{s},t_{i})f'_{s}(\mathbf{v}_{sf},t_{i}+\Delta t|\mathbf{v}_{sf}-\Delta\mathbf{v}_{s},t_{i})d^{3}\Delta\mathbf{v}_{s}.$ (S62)

Equation (S62) shows the total density of a specific particle at the time of$t_{i}+\Delta t$. Given that in a plasma region, most Coulomb collisions have little effect on the velocity of a moving particle, we can expand equation (S62) around the small values of$\Delta\mathbf{v}_{s}$ as:

$\begin{matrix} f_{s}(\mathbf{v}_{sf},t_{i})+\sum_{n=1}^{5} \frac{\left(\Delta t\right)^{n}}{n!}\frac{\partial^{n}f_{s}}{\partial t^{n}}|_{t=t_{i}}=\int f_{s}(\mathbf{v}_{sf},t_{i})f'_{s}(\mathbf{v}_{sf}+\Delta\mathbf{v}_{s},t_{i}+\Delta t|\mathbf{v}_{sf},t_{i})d^{3}\Delta\mathbf{v}_{s} \\ \begin{matrix} & & & + \end{matrix}\Delta t\sum_{n'=1}^{5} \frac{\left(-1\right)^{n'}}{n'!}\frac{\partial^{n'}}{\partial v_{s1}...\partial v_{sn'}}\times\left(\left\langle\Delta\mathbf{v}_{s1}...\Delta\mathbf{v}_{sn'}\right\rangle f_{s}(\mathbf{v}_{sf},t_{i})\right), \end{matrix}$ (S65)

where in equation (S65), the Taylor expansion is only given by the five terms to consider the significance of 1/lnΛ terms in strongly coupled plasmas. Moreover, having regard for the values of unity as the summation of all probabilities of particles velocity changes, we can calculate the expectation values of the velocity changes components,$\Delta\mathbf{v}^{n'}$by:

$\left\langle\left(\Delta\mathbf{v}_{sf}\right)^{n'}\right\rangle=\frac{1}{\Delta t}\int\left(\Delta\mathbf{v}_{s}\right)^{n'}f'_{s}(\mathbf{v}_{sf}+\Delta\mathbf{v}_{s},t_{i}+\Delta t|\mathbf{v}_{sf},t_{i})d^{3}\Delta\mathbf{v}_{s}.$ (S66)

In the second step, by assuming that the test particle, *s*, collides with the field particle,$s',$with the velocity$\mathbf{v}_{s'}$, and using the collision operator as:

$C^{ss'}=\frac{\partial f_{s}(\mathbf{v}_{sf},t_{i})}{\partial t_{i}}=\frac{\partial f_{s}(\mathbf{v}_{sf},t_{i})}{\partial t_{i}}+\mathbf{v}_{sf}.\frac{\partial f_{s}(\mathbf{v}_{sf},t_{i})}{\partial x_{i}}+\mathbf{a}_{sf}.\frac{\partial f_{s}(\mathbf{v}_{sf},t_{i})}{\partial v_{si}},$ (S67)

and equation (S65), we can present the general expression for the Fokker-Planck (FP) equation as:

$C^{ss'}=\frac{\partial f_{s}(\mathbf{v}_{sf},t_{i})}{\partial t_{i}}=\sum_{n'=1}^{5} \frac{\left(-1\right)^{n'}}{n'!}\frac{\partial^{n'}}{\partial v_{s1}...\partial v_{sn'}}\times\left(\left\langle\Delta\mathbf{v}_{s1}...\Delta\mathbf{v}_{sn'}\right\rangle f_{s}(\mathbf{v}_{sf},t_{i})\right),$ (S68)

where the odd and even values of$n'$determine the friction and diffusion coefficients, respectively. To derive the appropriate terms in equation (S68) as well as to receive a proper understanding of the governing transport processes among the different species moving relative to each other, we have followed the similar procedure performed in equations (S1-S58). Moreover, we have adopted the assumption on the energy exchange density and force density for the Maxwellian distribution function proposed recently by Baalrud and Daligault [18]. Similar to reference 18, we consider$\mathbf{v}$ and$\mathbf{v}'$as the initial velocities of colliding particles, while$\hat{\mathbf{v}}$and$\hat{\mathbf{v}}'$as the post-collision velocities. In this case equation (S68) can be interpreted as:

$\begin{matrix} C^{ss'}=\sum_{n'=1}^{5} \frac{\left(-1\right)^{n'}}{n'!}[\frac{\partial^{n'}}{\partial v_{s1}...\partial v_{sn'}}\times\left\langle\Delta\mathbf{v}_{s1}...\Delta\mathbf{v}_{sn'}\right\rangle f_{s}(\mathbf{v}_{sf},t_{i})) \\ \begin{matrix} \begin{matrix} & & \end{matrix} & & \end{matrix}-\frac{\partial^{n'}}{\partial v'_{s'1}...\partial v'_{s'n'}}\times\left\langle\Delta\mathbf{v}'_{s1}...\Delta\mathbf{v}'_{sn'}\right\rangle f_{s'}(\mathbf{v}'_{s'f},t_{i})], \end{matrix}$ (S69)

where each term of Boltzmann collision operator declares as:

(S70)

where **u** and$\sigma_{ss'}$regard as the relative velocity vector and the differential scattering cross-section, respectively. The transport coefficient for fluid equation is shown from velocity momentum as:

$\left\langle\Delta v\right\rangle^{s-s'}=\int d^{3}v\Delta v(\mathbf{v})C^{ss'},$ (S71)

where$\Delta v$ is the velocity-dependent function related to momentum, energy, continuity, etc. Substituting equation (S70) in equation (S71), we have:

(S72)

where$\Delta v^{\mu}$ is determined as:

$\Delta v^{\mu}=\frac{m_{s'}}{m_{s}+m_{s'}}\Delta u^{\mu},$ (S73)

(S74)

where$e_{i=\mu,\nu,..}$are the unit vectors in an arbitrary orthogonal coordinate system. Therefore, we can consider the generalized form of$\Delta\mathbf{u}_{L}$as:

$\Delta\mathbf{u}_{L}^{1}=-2u\sin^{2}\left(\theta/2\right),$ (S75)

(S76)

(S77)

where the superscripts 1-3 show the directions of the coordinate axes. For the scattering cross-section, one can write the$\left\{\Delta v^{\mu}\right\}$integrals using the impact parameter *b* and the scattering angle *θ*, which can be determined as equation (S78):

(S78)

where *r_0_* is a distance of closest approach, andregards as the interaction potential energy. To calculate the mean potential force, one can suppose in an elementary volume element$d\mathbf{r}d\mathbf{r}'$around$\left(\mathbf{r},\mathbf{r}'\right)$, two particles are found by the probability of [7]:

$\rho^{(2)}(\mathbf{r},\mathbf{r}')=\left\langle\sum_{i=1}^{N} \sum_{j=1,j\neq i}^{N} \delta\left(\mathbf{r}-\mathbf{r}_{i}\right)\delta\left(\mathbf{r}'-\mathbf{r}_{j}\right)\right\rangle$ (S79)

$$\begin{matrix} & & & = \end{matrix}N(N-1)\left(\int\exp(-\frac{\sum_{i,j} v(|\mathbf{r}_{i}-\mathbf{r}_{j}|)}{k_{B}T})d\mathbf{r}_{N}\right)^{-1}.\int\exp(-\frac{\sum_{i,j} v(|\mathbf{r}_{i}-\mathbf{r}_{j}|)}{k_{B}T})d\mathbf{r}_{3}...d\mathbf{r}_{N},$$

where$v$regards as the bare interaction potential energy. Note that regardless of the position of other particles, the contribution of all velocities is ignored in equation (S79). Considering$ng(r)=\rho^{(2)}(r)/n$ as an average radial density distribution around individual particles in a homogeneous and isotropic plasma, one can calculate the potential of mean force,by equations (S80) and (S81):

(S80)

(S81)

The critical part of calculating equation (S81) is to approximate the pair distribution function, *g*(*r*) value. In this paper, we use the hypernetted-chain (HNC) approximation, which contains both correlation and screening effect self-consistently [7]. Similar to reference 7, by using HNC method, we first consider *g*(*r*) as the coupled set of equations:

$g\left(\mathbf{r}\right)=\exp[-v(\mathbf{r})/k_{B}T+h\left(\mathbf{r}\right)-c\left(\mathbf{r}\right)],$ (S82)

$\hat{h}(\mathbf{k})=\hat{c}\left(\mathbf{k}\right)\left[1-n\hat{h}(\mathbf{k})\right],$ (S83)

where$\hat{h}(\mathbf{k})$regards as the Fourier transform of$h(\mathbf{r})$, and$h(r)=g\left(r\right)-1$is the pair correlation function. By substituting equation (S82) in equation (S80), we can calculate the effective potential value appeared between two particles at a distance *r* apart as the summation of the total bare interaction and a term determined the effect of the surrounding medium of the latter, in the HNC approximation:

(S84)

Using equation (S84), we can find the Debye screened potential, *c*(*r*) in HNC approximation as:

(S85)

Note that in the weakly coupled regime one can find; thus, equation (S85) approximately reduces to$c(r)\simeq-v(r)/k_{B}T$, which can connate. In contrast, the pair-correlation function can be reduced to the screened Coulomb potential,$v_{sc}(r)$, if the bare interaction is considered Coulombic,$\hat{v}\left(\mathbf{k}\right)=4\pi Z_{s}Z_{s'}e^{2}/k^{2}$. In this case, we would have:

$v_{sc}(r)=-h\left(r\right)=\frac{Z_{s}Z_{s'}e^{2}}{r}\exp\left(-\frac{r}{\lambda_{D}}\right),$ (S86)

where$\lambda_{D}=\sqrt{k_{B}T/4\pi Z_{s}Z_{s'}e^{2}n}$ is the Debye length. Following equations (S59-S86), we calculated the average dynamical friction (equations (S87), (S89), and (S91)), and tensor terms of velocity diffusion (equations (S88) and (S90)) as below:

$\frac{\left\langle\Delta\mathbf{v}_{ss'}\right\rangle}{\Delta t}=-\frac{m_{ss'}}{m_{s}}\int d^{3}v_{s}d^{3}v'_{s'}f_{s}\left(\mathbf{v}_{s}\right)f_{s'}\left(\mathbf{v}'_{s'}\right)\frac{\mathbf{u}}{u^{3}}u^{4}\bar{\sigma}_{ss'}^{\left(l\right)},$ (S87)

$\frac{\left\langle\Delta\mathbf{v}_{ss'}\Delta\mathbf{v}_{ss'}\right\rangle}{\Delta t}=\left(\frac{m_{ss'}}{m_{s}}\right)^{2}\int d^{3}v_{s}d^{3}v'_{s'}f_{s'}\left(\mathbf{v}_{s'}\right)f_{s}\left(\mathbf{v}'_{s}\right)\frac{u^{2}I-\mathbf{uu}}{u^{3}}u^{4}\bar{\sigma}_{ss'}^{\left(l\right)}\left(1+\frac{2}{5\ln\bar{\Xi}_{ss'}^{(l,k)}}\right),$ (S88)

$\frac{\left\langle\Delta\mathbf{v}_{ss'}\Delta\mathbf{v}_{ss'}\Delta\mathbf{v}_{ss'}\right\rangle}{\Delta t}=-\left(\frac{m_{ss'}}{m_{s}}\right)^{3}\int d^{3}v_{s}d^{3}v'_{s'}f_{s}\left(\mathbf{v}_{s}\right)f_{s'}\left(\mathbf{v}'_{s'}\right)\left[\frac{\mathbf{u}}{u}I-\frac{\mathbf{uuu}}{u^{3}}\right]u^{4}\bar{\sigma}_{ss'}^{\left(l\right)}\frac{1}{\ln\bar{\Xi}_{ss'}^{(l,k)}},$ (S89)

$\begin{matrix} \frac{\left\langle\Delta\mathbf{v}_{ss'}\Delta\mathbf{v}_{ss'}\Delta\mathbf{v}_{ss'}\Delta\mathbf{v}_{ss'}\right\rangle}{\Delta t}=\left(\frac{m_{ss'}}{m_{s}}\right)^{4}\int d^{3}v_{s}d^{3}v'_{s'}f_{s}\left(\mathbf{v}_{s}\right)f_{s'}\left(\mathbf{v}'_{s'}\right) \\ \begin{matrix} \begin{matrix} & & & \end{matrix} & & & \end{matrix}.\left[u \mathbf{u}+\frac{u^{2}}{u^{3}}I-\frac{\mathbf{uuu}}{u^{3}}\right]u^{4}\bar{\sigma}_{ss'}^{\left(l\right)}\frac{1}{\ln\bar{\Xi}_{ss'}^{(l,k)}}, \end{matrix}$ (S90)

$\begin{matrix} \frac{\left\langle\Delta\mathbf{v}_{ss'}\Delta\mathbf{v}_{ss'}\Delta\mathbf{v}_{ss'}\Delta\mathbf{v}_{ss'}\Delta\mathbf{v}_{ss'}\right\rangle}{\Delta t}=-\left(\frac{m_{ss'}}{m_{s}}\right)^{5}\int d^{3}v_{s}d^{3}v'_{s'}f_{s}\left(\mathbf{v}_{s}\right)f_{s'}\left(\mathbf{v}'_{s'}\right) \\ \begin{matrix} \begin{matrix} \begin{matrix} & & \end{matrix} & & & \end{matrix} & & & . \end{matrix}\left[\frac{\mathbf{u}}{u}I-2u^{2} \mathbf{u}+\frac{\mathbf{uu}}{u^{2}}-\frac{\mathbf{uuu}}{u^{3}}\right]u^{4}\bar{\sigma}_{ss'}^{\left(l\right)}\frac{1}{\ln\bar{\Xi}_{ss'}^{(l,k)}}. \end{matrix}$ (S91)

It should be noted our calculated equations (S87-S91) can be interpreted in terms of the base integral form of:

$I_{ss'}^{(l,k)}=2\sqrt{2}\sigma_{0}\ln\Lambda_{ss'}\sqrt{\pi(\frac{k_{B}T_{s}}{m_{s}}+\frac{k_{B}T_{s'}}{m_{s'}})}\int_{0}^{\pi} d\theta\sin\theta\left(1-\cos^{l}\theta\right),$ (S92)

where by considering$\bar{v}_{ss'}^{4}=4\left(T_{s}/m_{s}+T_{s'}/m_{s'}\right)^{2}$as the average velocity, the generalized Coulomb logarithm,$\ln\bar{\Xi}_{ss'}^{(l,k)}$, associated with$I_{ss'}^{(l,k)}$, has been defined as:

$\ln\bar{\Xi}_{ss'}^{(l,k)}=\frac{1}{2}\int_{0}^{\infty} d\xi\xi^{2k+3}\frac{\exp(-\xi^{2})\bar{\sigma}_{ss'}^{\left(l\right)}}{\sigma_{0}},$ (S93)

where,

$\bar{\sigma}_{ss'}^{(l)}=2\pi\int_{0}^{\infty} db b\left[1-\cos^{l}\theta\right],$ (S94)

in which$\xi=\left|\mathbf{u}\right|/\bar{v}_{ss'},$ $\sigma_{0}=\left(\pi Z_{s}^{2}Z_{s'}^{2}e^{4}\right)/\left(m_{ss'}^{2}\bar{v}_{ss'}^{4}\right),$ $m_{ss'}=m_{s}m_{s'}/\left(m_{s}+m_{s'}\right)$and$\bar{\sigma}_{ss'}^{l}$is the *l^th^* momentum-transfer cross-section with the scattering angle of *θ* and the impact parameter of *b*. Considering a spherical coordinate system$\left(\hat{e}_{z}=\Delta\hat{\mathbf{V}}\right)$, corresponded to the velocity of:

(S95)

we can define$\Delta\bar{\mathbf{V}}_{ss'}\equiv\Delta\bar{\mathbf{v}}_{ss'}/\bar{v}_{ss'}$. Comparing our calculated equations (S87-S91), one can obtain an improved coefficient,$1+2/(5\ln\bar{\Xi}_{ss'}),$ in the second term of Taylor expansion which clearly shows the dependence of the second term on the Coulomb logarithm in high-order terms of expansion. When the test particle, *s*, collides with the background moving particle,$s'$, we can calculate the rate of energy loss of the test particle as$dE_{s}/dt=(m_{s}/2)(\left\langle\Delta\mathbf{v}_{ss'}^{2}\right\rangle/\Delta t),$ which can be determined from the coefficients calculated in equations (S87-S91) as below:

$\frac{dE_{s}}{dt}\cong\frac{m_{s}}{2}\mathbf{v}_{s}(\sum_{n'=1}^{5} \frac{1}{\mathbf{v}_{s}^{n'-1}}\frac{\left\langle\left(\Delta\mathbf{v}_{ss'}\right)^{n'}\right\rangle}{\Delta t}).$ (S96)

1. [mahsamehrangiz.phys@gmail.com](mailto:mahsamehrangiz.phys@gmail.com) [↑](#footnote-ref-1)
2. [skhoshbinfar@guilan.ac.ir](mailto:skhoshbinfar@guilan.ac.ir) [↑](#footnote-ref-2)
